# A systematic review of human studies assessing the health effects of unburned kerosene-based jet fuels and products across diverse populations and settings

**DOI:** 10.1186/s12940-026-01287-7

**Published:** 2026-03-16

**Authors:** Veronica Carvajal, Brandon Ng, Nicole Rosendaal, Catherine M. Pirkle

**Affiliations:** Department of Public Health Sciences, University of Hawaiʻi at Mānoa, 1960 East-West Rd Honolulu, Hawaiʻi, 96822 USA

**Keywords:** Petroleum hydrocarbons, Kerosene, Jet fuel, Environmental health, Epidemiology, Human health

## Abstract

**Objective:**

Petroleum hydrocarbons are significant environmental health concerns globally, due to their extensive use and environmental release, including water contamination events such as the 2021 Red Hill jet fuel spill. Research on kerosene and kerosene-based jet fuels is limited, with prior reviews often focused on occupational and post-combustion exposures. No previous systematic review has focused specifically on pre-combustion exposures. Thus, we systematically reviewed evidence of human health impacts associated with these raw fuels across all exposure settings and population groups.

**Materials and methods:**

Search strategies and eligibility criteria were developed and executed following PRISMA reporting guidelines and informed by OHAT procedures for systematic reviews. PubMed and Web of Science databases were searched (2017–2024) for eligible literature. Data extraction, analyses, quality assessment and risk of bias appraisals were conducted. Four reviewers participated in these processes.

**Results:**

A total of 28 articles were included. Respiratory outcomes were frequently observed following acute kerosene ingestion, whereas neurological outcomes were common with chronic occupational, dermal and inhalation exposure to jet fuel. Evidence of gastrointestinal and dermal outcomes were also documented. Current literature did not allow outcomes with long latency to be assessed. Limited analytical studies and data heterogeneity challenge the ability to establish robust conclusions.

**Conclusion:**

Considering the global health implications of kerosene and kerosene-based jet fuel, and recent water contamination events, this review is novel and timely in its focus on unburned fuel. Current evidence on the human health consequences of these fuels is limited. We thus provide recommendations for future studies, including exposure assessment methods, study designs, and prioritization of underrepresented populations.

**Supplementary Information:**

The online version contains supplementary material available at 10.1186/s12940-026-01287-7.

## Background

Understanding health effects associated with raw jet fuel is increasingly urgent in light of recent water contamination events. In November 2021, a spill of 5,542 gallons of the kerosene-based jet propellant 5 (JP-5) at the U.S. Navy’s Red Hill Bulk Fuel Storage Facility on Oʻahu, Hawaiʻi contaminated the Navy-managed system supplying drinking water to Joint Base Pearl Harbor Hickam and surrounding communities [1]. Approximately 93,000 individuals, ranging in age from less than a year to over 65 years of age [2, 3] were exposed to the jet fuel-contaminated water [3]. This event garnered extensive global attention [4–6], resulting in the defueling of the facility and ongoing legal battles covered extensively by the media [7–9]. More recently, in Bucks County, Pennsylvania, a jet fuel leak was confirmed in January 2025, in which JP-8 emanated from Sunoco’s Twin Oaks pipeline and tainted well water [10, 11]. The leak is believed to have been ongoing for at least 16 months [12, 13]. These events highlight jet fuel exposures in the general population, specifically through ingestion, topics that are both understudied.

Jet fuels are frequently released into the environment and can disperse through air, soil, and water, presenting an environmental health risk to communities in proximity to these releases [14]. In 2017, the Agency for Toxic Substances and Disease Registry (ATSDR) [14] published a toxicological profile consolidating the existing research on potentially-associated health effects of jet fuel. While their scope was not exclusive, the human-specific data available were derived primarily from occupational reports. Additional reviews specific to human health outcomes have been published since; however, the focus of these studies were restricted to occupational and/or military settings. As a result, much of the evidence on the health outcomes associated with jet fuel exposure in humans is based on inhalation and dermal exposures among men of working age [15–17]. Many of the occupational studies conducted thus far have not distinguished between pre- or post-combustion exposures, ultimately limiting the ability to attribute toxicological outcomes to specific forms of the fuel and their unique chemical compositions. Similarly, prior systematic reviews evaluating health implications of kerosene use (the bulk component of most jet fuels) [14, 18] have focused on household air pollution (HAP), thereby limiting the knowledge of health implications associated with raw, unburned fuel specifically [19, 20]. As a result of the limited scope of most research on jet fuels and its primary component, there is a critical research gap on the potential health outcomes of residential, community and oral exposures, particularly in sub-populations including women, children, and older adults. Thus, a systematic review is warranted to comprehensively identify and evaluate the available evidence on the health effects of exposure to raw jet fuel and kerosene.

### Total petroleum hydrocarbons as global environmental health concern

Petroleum hydrocarbons (PH) comprise hundreds of crude oil derived chemical compounds. They are described as the most prevalent environmental contaminants globally [21–23] due to their extensive anthropocentric uses in transportation, heating, and industry [24, 25]. Global petroleum consumption reached 97.3 million barrels per day (b/d) in 2021 [26]. Total petroleum hydrocarbons (TPH) refer to the combined measurable concentration of PHs in a sample [24] These compounds enter the environment through the refinement of crude oil, operational failures, leakages, spills, and as byproducts from commercial or private uses, ultimately contaminating water, soil and air [24, 25]. When released into water, lighter TPH fractions may float, while heavier fractions accumulate in sediment, potentially impacting wildlife who inhabit affected areas, and those that feed on them [24, 25] with potential human health implications following consumption of these organisms. In soil, TPHs can seep from the source area, evaporate into air, and/or dissolve into groundwater [24]. In other instances, TPH compounds may enter groundwater directly [1].

Considering that TPHs commonly spread from the area in which they are released, people may come into contact with these compounds even when at distance from the pollution site [25]. TPHs may enter the body when inhaled, ingested, or in contact with the skin [24], and impact various organ systems depending on how they are metabolized and distributed [24]. The resulting health effects are dependent on the nature of the compounds themselves, as well as the duration and dose of exposure [24]. While the toxicity of particular fractions such as benzene, toluene, ethylbenzene and xylenes are well described, the human health effects associated with exposure to more complex fuel mixtures [24, 25], such as jet fuel and kerosene, are less understood despite their extensive use and frequent release into the environment.

### Kerosene & jet fuel: composition, use and limitations of existing research

In 2022, daily petroleum consumption averaged approximately 20.3 million b/d in the United States (U.S.) alone, for industry, residential and commercial purposes [26]. More than half of this use (67%) went to transportation including the 1.6 million barrels of kerosene-type jet fuel that accounted for 8% of total daily petroleum consumption [26]. In contrast, kerosene as a standalone product was consumed much less (0.004 million b/d) [26].

Kerosene (commonly known as fuel oil no. 1, paraffin, paraffin oil, lamp oil) is a colourless to yellow appearing petroleum-derived oil used as household fuel for heaters, lamps, furnaces, diesel and tractor engines, as well as in lubricants and pesticides [24, 25, 27, 28]. In low-and-middle-income countries (LMIC) kerosene is often used as fuel for cooking and lighting, contributing to air pollution that has been associated with various adverse health outcomes [20, 27]. Thus, studies examining the health effects of kerosene use in the home generally assess outcomes related to combustion-specific byproducts, without consideration of potential risks of exposure to raw fuel. Adverse human health outcomes of exposure to kerosene pre-combustion are documented; however, the available evidence is limited and stems primarily from reports of accidental ingestion in children of LMIC [15, 24].

While residential usage has steadily declined in higher income countries, globally, kerosene continues to be used frequently in commercial and military aviation, predominantly as jet fuel [25, 27]. Although a small amount of jet fuel is produced from oil sands, the vast majority is kerosene-based [14, 18]. These kerosene-type jet fuels contain varying proportions of kerosene itself, along with performance additives, including antioxidants and other agents to prevent icing, static buildup, corrosion and bacterial growth [24, 25]. Two major kerosene-based jet fuels are used in commercial aviation: Jet A and Jet A-1. Jet A-1 has a lower maximum freezing point suitable for international flights. Jet A is used commercially for flights within the continental U.S. and by the U.S. Air Force [14]. Military jet propellants (JP) include JP-4, 5, 6, 7, 8 [14], each differing in intended application and chemical composition [25]. Of these, JP-5 and JP-8 are the two kerosene-based aircraft fuels used currently by the U.S. military and contain approximately 99.5% kerosene [14, 25]. Due to the increased safety associated with its higher flash point (i.e. the lowest temperature at which a liquid can be ignited when an ignition source is present), JP-5 is used by the U.S. Navy aboard aircraft carriers [14]. JP-8 is chemically similar to Jet A-1, but with military-specific performance additives, and is the most widely used jet fuel by the U.S. military in both air and ground applications [14, 15].

Jet fuel is the largest chemical exposure among U.S. military personnel, and a frequent occupational exposure in non-military settings, particularly for those involved in fueling, transporting and routine maintenance of aircraft [14, 15]. These individuals are at an increased risk of exposure, particularly through dermal absorption and inhalation of both pre- and post- combustion fuel forms [14]. This elevated risk is reflected in current research, where the limited number of human studies assessing jet fuel associated health outcomes are predominantly focused on occupational exposures [14–17]. Researchers, however, recognize that the general population residing in close proximity to military bases and airports are at increased risk of exposure to jet fuels and toxic effects from them [14, 15, 29].

Jet fuels (including Jet A, JP-5 and JP-8) often enter the environment during routine processes such as storage, handling and transportation, in-flight jettisoning, as well as accidental spills or leaks [14]. Like other TPHs, jet fuel can migrate from the pollution site, with the potential to penetrate soil, enter groundwater, and/or be carried by the atmosphere following volatilization [14].

### Objective

Considering the widespread and frequent release of jet fuel into the environment, the historical focus on males in occupational settings and the limited understanding of health effects associated with pre-combustion jet fuel exposure in humans, we conducted a systematic review designed to capture information on these gaps. Given that kerosene constitutes approximately 99.5% of jet fuel, its inclusion in this review enables examination of this toxicant across a broader range of exposure routes and populations that would remain unassessed with a focus on jet fuel alone. As the literature on jet fuel primarily involves inhalation and dermal contact among occupationally exposed men of working age - with little delineation between pre- versus post-combustion exposures - incorporating kerosene also allows for an evaluation of potential health effects linked to ingestion, in additional contexts and demographic groups, to raw, unburned fuel.

Thus, this review aims to systematically evaluate human studies that assess the association between oral, dermal and inhalation exposures to pre-combustion kerosene-based jet fuel, as well as other kerosene products, on health outcomes across all exposure settings and population groups.

## Methods

We structured our research question following the PECO framework (Population, Exposure, Comparator, Outcome) as summarized in Additional file 1. It was:*“What are the known and/or potential health effects of exposure (oral*,* dermal and inhalation) to pre-combustion forms of kerosene-based jet fuel*,* and other kerosene products*,* in humans across all exposure settings and population groups?”.* Our search protocol for the review was registered in The International Prospective Register of Systematic Reviews (PROSPERO) (CRD42025645179). In creating and executing the methodology for this study, Preferred Reporting Items for Systematic reviews and Meta-Analyses (PRISMA) guidelines [30] were followed, and the Office of Health Assessment and Translation (OHAT) procedures for Systematic Reviews [31] were applied in evidence identification, data extraction and synthesis, as well as a risk of bias appraisals.

### Assessment of existing and ongoing systematic reviews

Cochrane, Epistemonikos, and PROSPERO were searched for any published or ongoing systematic reviews evaluating the research topic of the current study: health effects of pre-combustion forms of kerosene-based jet fuel or other kerosene products, in humans across all exposure settings and population groups. None were found. The search strategy and results are provided in Additional file 2.

### Search strategy and eligibility criteria

In collaboration with a university public health librarian, the search strategy was designed with the following structure: (Jet fuel & Kerosene terms) AND (Health effects terms). Two databases (PubMed and Web of Science) were utilized to conduct the literature searches, which is consistent with literature on the subject [32, 33]. Detailed search strategies (including exposure and health outcome terms) as well as limits (publication years and article types) utilized for each database search can be found in Table 1 (search conducted February 3, 2025). Health effect terms were screened for added value; those terms that did not pull additional results for the search strategy were not included. These terms can be found in Additional file 3. Additionally, we conducted retrospective citation tracing of included publications.

**Table 1:** Databases and Search Strategies Used. This table lists the databases searched during the literature review and the specific search strategies applied in each, including date ranges and filters where applicable

Database	Search terms	Limitations	Output
PubMed	((“JP5 jet fuel” [Supplementary Concept] OR “S-8 fuel” [Supplementary Concept] OR “JP8 aviation fuel” [Supplementary Concept] OR “Kerosene“[Mesh] OR kerosene[Title/Abstract] OR “jet propellant“[Title/Abstract] OR “jet propellants“[Title/Abstract] OR “aviation fuel“[Title/Abstract] OR “aviation fuels“[Title/Abstract] OR “jet fuel“[Title/Abstract] OR “jet fuels“[Title/Abstract] OR “turbine fuel“[Title/Abstract] OR “turbine fuels“[Title/Abstract] OR “jet propulsion fuel“[Title/Abstract] OR “jet propulsion fuels“[Title/Abstract] OR “Jet A“[Title/Abstract] OR “Jet A-1“[Title/Abstract] OR “Jet B“[Title/Abstract] OR “JP-1“[Title/Abstract] OR “JP1“[Title/Abstract] OR “JP-2“[Title/Abstract] OR “JP2“[Title/Abstract] OR “JP-3“[Title/Abstract] OR “JP3“[Title/Abstract] OR “JP-4“[Title/Abstract] OR “JP4“[Title/Abstract] OR “JP-5“[Title/Abstract] OR “JP5“[Title/Abstract] OR “JP-6“[Title/Abstract] OR “JP6“[Title/Abstract] OR “JP-7“[Title/Abstract] OR “JP7“[Title/Abstract] OR “JP-8“[Title/Abstract] OR “JP8“[Title/Abstract] OR “zip fuel“[Title/Abstract] OR “zip fuels“[Title/Abstract] OR “JPTS“[Title/Abstract] OR “Jet Propellant Thermally Stable“[Title/Abstract] OR “TS-1“[Title/Abstract])) AND ((“Diseases Category“[Mesh] OR “Psychiatry and Psychology Category“[Mesh] OR depression[Title/Abstract] OR “analysis“[Subheading] OR “epidemiology“[Subheading] OR “toxicity” [Subheading] OR “Toxicology“[Mesh] OR “injuries“[Subheading] OR “etiology“[Subheading] OR “Pathologic Processes“[Mesh] OR “physiology“[Subheading] OR Health[Title/Abstract] OR “health effect“[Title/Abstract] OR “health effects“[Title/Abstract] OR “adverse effect“[Title/Abstract] OR “adverse effects“[Title/Abstract] OR “side effect“[Title/Abstract] OR “side effects“[Title/Abstract] OR disease[Title/Abstract] OR morbidit*[Title/Abstract] OR mortalit*[Title/Abstract] OR incidence[Title/Abstract] OR prevalence[Title/Abstract] OR symptom[Title/Abstract] OR heart[Title/Abstract] OR skin[Title/Abstract] OR dermal[Title/Abstract] OR renal[Title/Abstract] OR cancer[Title/Abstract] OR lung[Title/Abstract] OR exposure[Title/Abstract] OR reproductive[Title/Abstract] OR genetic[Title/Abstract] OR imbalance[Title/Abstract] OR breath*[Title/Abstract] OR rash*[Title/Abstract] OR metabol*[Title/Abstract] OR pain[Title/Abstract] OR coordination[Title/Abstract] OR memory[Title/Abstract] OR fatigue[Title/Abstract] OR weak*[Title/Abstract]))	Publication date: 2017–2024	862
Web of Science	(AB=((“Jet Propellant” OR “Jet Propellants” OR “Aviation Fuel” OR “Aviation Fuels” OR “Jet Fuel” OR “Jet Fuels” OR “turbine fuel” OR “turbine fuels” OR “jet propulsion fuel” OR “jet propulsion fuels” OR “Jet A” OR “Jet A-1” OR “Jet B” OR “JP-1” OR “JP1” OR “JP-2” OR “JP2” OR “JP-3” OR “JP3” OR “JP-4” OR “JP4” OR “JP5 jet fuel” OR “JP-5” OR “JP5” OR “JP-6” OR “JP6” OR “JP-7” OR “JP7” OR “S-8 fuel” OR “JP-8” OR “JP8” OR “JPTS” OR “Jet Propellant Thermally Stable” OR “TS-1” OR “zip fuel” OR “zip fuels” OR kerosene))) AND AB=((“adverse effect” OR “adverse effects” OR “side effect” OR “side effects” OR “long term effect” OR “long term effects” OR “acute effect” OR “acute effects” OR exposure OR health OR disease* OR illness* OR disorder OR mortalit* OR incidence OR prevalence OR “quality of life” OR depression OR poison* OR toxic* OR heart OR digest* OR reproduct* OR immun* OR metaboli* OR respirat* OR asthma OR bronchitis OR lung OR breath* OR skin OR renal OR cancer* OR memory OR eye* OR gene OR genetic OR mutation OR hormon* OR vision OR confusion OR diarrhea OR imbalance OR pregnancy OR male OR female OR enzymes))	Document type: Article, Early Access Publication date: 2017-01-01 to 2024-12-21	1,354

For an article to be eligible for inclusion in this review, it had to: (a) assess the association between oral, dermal and/or inhalation exposures to pre-combustion forms (i.e. before fuel is burned) of kerosene-based jet fuel or other kerosene products, and health related effects in humans; studies focused solely on animal models or post-combustion forms of jet fuel were excluded; (b) be peer reviewed and an original study; grey literature, letters to editors, editorials and reviews were excluded; (c) be published between 2017 and 2024 in English, French, Dutch, Spanish or Portuguese; (d) be experimental, descriptive or observational in design, including but not limited to randomized control trials, case studies, case-control and cohort studies; (e) conduct investigations in-vivo; studies conducted ex-vivo were excluded. The publication period was selected to build upon the evidence consolidated by the U.S. ATSDR in their peer-reviewed toxicological profile on jet fuels published in 2017 [14]. No limitations were placed on the region in which the studies were conducted. While texts were eligible for inclusion if in English, French, Dutch, Spanish or Portuguese, searches were conducted in English.

**Fig. 1:**
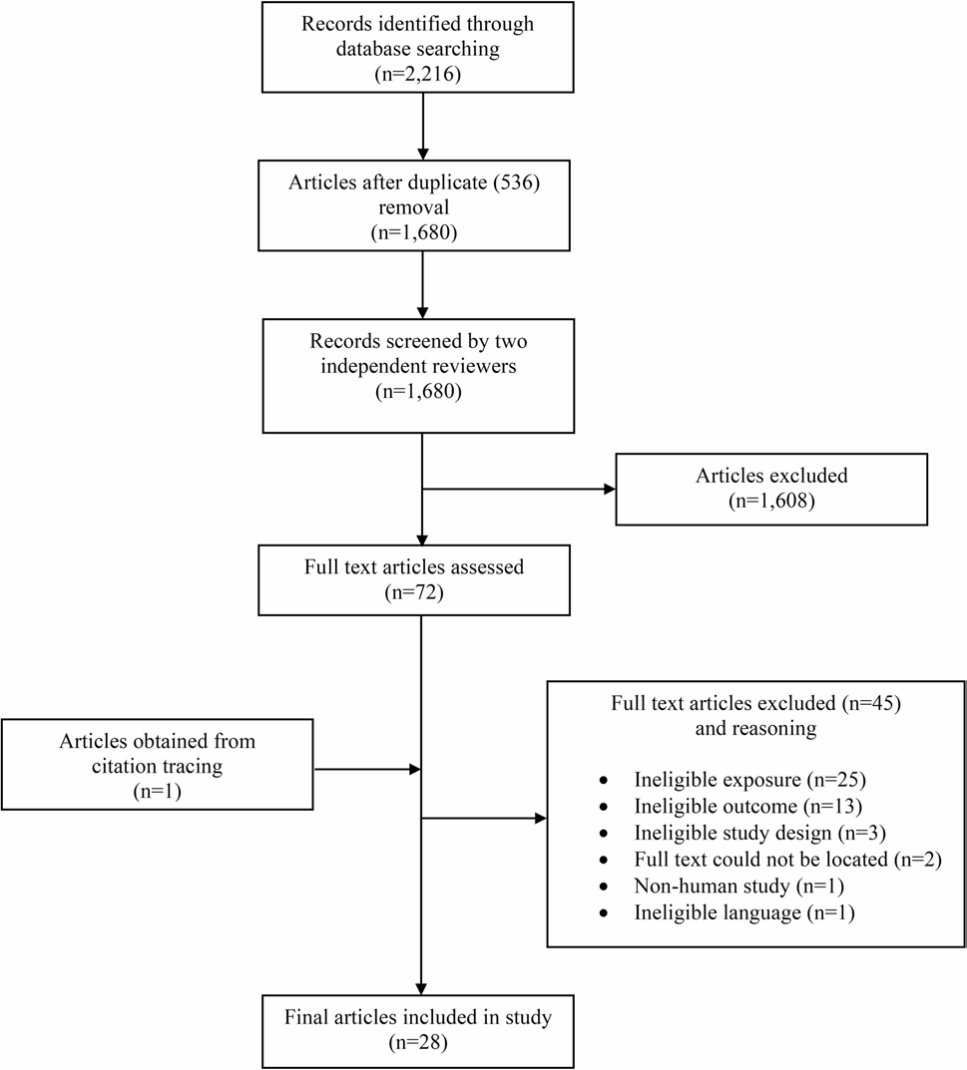
Article Selection Process. This figure summarizes the flow of studies through the screening phases of the systematic review, including reasons for exclusion and final numbers included

Articles retrieved from the database searches were uploaded to the Rayyan platform for de-duplication, screening and selection of articles [34]. Two reviewers (VC and BN) conducted blind independent screening of all titles and abstracts, as well as full texts. Reviewers met after each stage of the screening process to discuss any conflicting decisions on article eligibility. A third reviewer (CP) was consulted when consensus could not be reached between the first and second reviewers. The article selection process can be found in Fig. 1.

### Data extraction

Data extracted from included articles consisted of: first author, publication year, country in which the study was conducted, study design, sample size, age and sex of participants, type, route and setting of exposure, exposure assessment methods, health outcomes assessed, outcome assessment methods, statistical models, measures of effect, covariates adjusted for in statistical analyses, and main findings applicable to the research question of this review. Study designs were recorded as stated by the original authors. Each included study was data extracted blindly and independently by two reviewers (VC and BN). Any discrepancies in extracted data was resolved through discussion to ensure the accuracy and consistency of findings. If data were missing from a given study, it was recorded as “Information not provided” and accounted for when conducting quality assessment of included studies. Extracted data for each study can be found in Table 2.

**Table 2:** Data Extracted from Included Studies. This table summarizes key information from included studies, such as authors, publication year, study design, participant characteristics, exposure and outcome measures, statistical analyses and main findings

Author & year, study country & design	Sample size (*N*), participants’ sex & age	Exposure type, route, setting	Exposure assessment methods	Outcome assessed & method	Statistical models & measures of effect	Covariates adjusted for	Main findings
Areprekumor et al., 2024 [35] Nigeria Cross-sectional	*N* = 14 Female (41%) Male (59%) Age: <5, 5–10, > 10 y	Kerosene Ingestion, inhalation Residential, workplace, other	Self reported, no measurement conducted	Clinical presentation Analysis of hospital records	Descriptive statistics None	None	Cough, fever, respiratory distress, vomiting, loss of consciousness, convulsion, mortality following severe aspiration pneumonia
Bateganya & Nakanjako, 2023 [36] Uganda & Kenya Descriptive Ethnographic	*N* = 48 Female (50%) Male (50%) Age: 10–17 y	Aviation Fuel Inhalation Community	Self reported, no measurement conducted	Side effects of health-seeking behaviours In-depth interviews, focus group discussions	None	None	Loss of appetite, early death
Benítez Riesco et al., 2024 [37] NR (author affiliation: Spain) Case report	*N* = 1 Male Age: 47 y	Kerosene (sailboat engine lubricant, kerosene main component) Dermal Residential	Self reported, no measurement conducted	No predefined outcome assessed Clinical evaluation, diagnostic tests	N/A	N/A	Proctalgia, perianal inflammation, anal ulcers Diagnosis: rectal burns injury from kerosene exposure
Chattopadhyay et al., 2021 [38] NR (author affiliation: India) Case report	*N* = 1 Female Age: 25 m	Kerosene Ingestion Residential	Self reported, no measurement conducted	No predefined outcome assessed Clinical evaluation, diagnostic tests	N/A	N/A	High fever, vomiting, aspiration pneumonitis with decreased air entry to right lung Diagnostic tests: decreased air entry to right lung, patchy air space consolidation in right lower zone of lung, moderate pleural effusion on right side with collapse, loculated collection in right middle and lower lobe with pleural thickening, pus pockets found in middle and lower lobe of right lung
Contestable, 2017 [39] NR (clinic on U.S. Navy aircraft carrier) Case report	*N* = 1 Male Age: 19 y	Jet Fuel (JP-5) Dermal Occupational (Military)	Self reported, no measurement conducted	No predefined outcome assessed Clinical evaluation	N/A	N/A	Worsening, mildly pruritic rash, erythematous, edematous, hyperkeratotic plaques with cracking, weeping, and crusting Diagnosis: contact dermatitis
Dayasiri et al., 2017 [40] Sri Lanka Cross-sectional	*N* = 196 Female (40%) Male (60%) Age: 9 m − 12 y	Kerosene Ingestion (98%), inhalation (2%) Residential	Self reported, no measurement conducted	Symptom profiling Hospital records	No statistical models stated None, proportions	None	Respiratory symptoms (cough/shortness of breath/wheezing), gastrointestinal (vomiting, nausea, abdominal pain), neurological symptoms (giddiness, drowsiness), cardiovascular symptoms, mortality (following aspiration pneumonia), aspiration pneumonia/chemical pneumonitis
*Dreisbach et al., 2022 [41] U.S. Cross-sectional	*N* = 48 Female (21%) Male (79%) Age: 19–29 y	Jet Fuel (JP-5) Dermal, inhalation Occupational (Military)	Personal air monitoring via passive samplers	Noise induced central auditory nervous system dysfunction mAIAD test for speech intelligibility in noise, speech intelligibility in quiet, localization of sound, recognition of sound, and detection of sound & audiologic testing	General linear modelling Group mean differences	None	No significant differences observed between audiometric thresholds & mAIAD scores in exposed vs. unexposed Unexposed group had less (but not significant) difficulty overall in listening situations Exposed group had consistent (but not significant) lower overall and subdomain scores on mAIAD (worse speech intelligibility in noise) compared to unexposed
Esashi et al., 2021 [42] NR (author affiliation: Japan) Case report	*N* = 1 Male Age: 85 y	Kerosene Inhalation, possible ingestion Residential	Self reported, no measurement conducted	No predefined outcome assessed Clinical evaluation, diagnostic tests	N/A	N/A	Dyspnea, cough, confusion (history of severe Alzheimer’s dementia) Diagnostic tests: bilateral coarse crackles, consolidation in bilateral lower lobes, double gastric fluid level and consolidation in right middle and left lower lobes Diagnosis: kerosene poisoning and aspiration pneumonitis
Fife et al., 2018 [43] NR (U.S. military bases) Case series	*N* = 3 Female (100%) Age: 37, 45 & 54 y	Jet Fuel (JP-8) Inhalation, dermal Occupational (Military)	Patient 1: self-reported, biomonitoring via blood samples (n-hexane, 3-methylpentane) Patient 2 & 3: self-reported, air monitoring, gas chromatography/mass spectrometry	No predefined outcome assessed Clinical evaluation, diagnostic tests	N/A	N/A	Dizziness (sensations of spinning, tilting, floating, disequilibrium and head fullness), headache, fatigue, moderately impaired equilibrium. mild right frontal hypoperfusion, imbalance, eye/skin irritation, cough, sinus congestion, chest tightness, irritability, depression, shortness of breath, palpitations, numbness, blurred vision, erratic heart beats, sneezing, recurrent sinus, upper respiratory tract, urinary tract and bladder infections Diagnostic tests: bilateral vestibular dysfunction with reduced gain, general impairment of equilibrium and predominant vestibular deficit pattern, hearing loss in right ear and borderline normal left-sided hearing, fall on Romberg test, difficulty walking
*Fuente et al., 2019 [44] Australia Cross-sectional	*N* = 57 Female: (12%) Male: (88%) Age: 23–64 y	Jet Fuel (JP-8) Inhalation, dermal and/or oral Occupational (Military)	Exposure category assigned based on task group and job history, self-reported exposure level, previous industrial assessments, evaluation by occupational hygienist	Peripheral and central auditory systems Tympanometry, pure-tone audiometry (1–12 kHz), DPOAEs, ABR, words-in-noise, compressed speech, dichotic digit test, pitch pattern sequence test, duration pattern sequence test and adaptive test of temporal resolution	Multivariate linear regression Adjusted mean differences	Age and noise level	Dose–response relationship, with poorer central auditory processing test scores (compressed speech, words-in-noise) observed at higher levels of jet fuel exposure Moderate and highly exposed groups presented with significantly worse hearing thresholds at 4 kHz in both ears and at 8 kHz in the right ear Significant association between exposure and average hearing threshold across frequencies (1–8 kHz) in the better ear Higher exposed personnel presented with significantly worse results on pure-tone thresholds DPOAE amplitudes, ABR wave V latency, and scores for both compressed speech and words-in-noise
Goenka et al., 2022 [45] India Case report	*N* = 1 Male Age: 62 y	Kerosene Ingestion NR	Self reported, no measurement conducted	No predefined outcome assessed Clinical evaluation, diagnostic tests	N/A	N/A	Fever, expectoration, decreased breath sounds with inspiratory rales and crackles bilaterally, cough, shortness of breath, left-sided pneumothorax Diagnostic tests: patchy infiltrates in bilateral lower zones, bilateral dependent dense consolidation and multiple pneumatoceles, ground-glass opacities, low attenuating (lipid-containing) opacities, thick pus drainage of lower lobe of left lung Diagnosis: lipoid pneumonia
Gupta et al., 2017 [46] India Case report	*N* = 1 Male Age: 28 y	Kerosene (Indigenous pesticide) Ingestion NR	Self reported, no measurement conducted	No predefined outcome assessed Clinical evaluation, diagnostic tests	N/A	N/A	Altered sensorium, tachypnoea, methaemoglobinaemia
Guss et al., 2020 [47] NR (author affiliation: U.S.) Case report	*N* = 1 Female Age: 13 y	Kerosene Dermal Community	Self reported, no measurement conducted	No predefined outcome assessed Clinical evaluation, diagnostic tests	N/A	N/A	Presentation mimicked mid palmar abscess, purulence, swelling, erythema, pain and stiffness in the hand Diagnostic tests: Continuous phlegmonous changes
Hara et al., 2018 [48] Japan Case report	*N* = 1 Male Age: 69 y	Kerosene (wood preservative) Inhalation Residential	Self reported, no measurement conducted	No predefined outcome assessed Clinical evaluation, diagnostic tests	N/A	N/A	Dyspnoea, delirium, tachycardic, tachypnoeic, labored breathing, burn injuries (face, neck, chest, upper arms and respiratory tract), encephalopathy, pneumonitis, dermatitis, acute respiratory distress syndrome Diagnostic tests: Bibasal coarse crackles, increased bilateral hilar shadow, infiltration in both lower lobes, return fluid resembled wood preservative Diagnosis: hydrocarbon pneumonitis due to inhalation of wood preservative containing kerosene
*Heaton et al., 2017 [49] NR (author affiliation & sample: U.S. military) Cohort	*N* = 73 Female (16%) Male (84%) Age: 18–43 y	Jet Fuel (JP-8) Inhalation Occupational (Military)	Personal air (THC, naphthalene) and urine (1-naphthol, 2-naphthol) sampling	Neurocognitive functioning Examiner-administered tests (Auditory Consonant Trigrams, Hooper Visual Organization Test, Hopkins Verbal Learning Test-Revised, Wechsler Adult Intelligence Scale III Digit Span Test, Grooved Pegboard) & computer-based tasks (ANAM4), Sleepiness Scale and Positive and Negative Affect Scale	Generalized linear modelling *β* coefficient	General intelligence (Shipley score), education, sex, age, years of Air Force service, enlisted status/rank, and race considered. Final model included Shipley score, sex, and years of AF service.	Increased performance with non-dominant hand on Grooved pegboard task Decreased performances in visual memory and motor speed
Kim et al., 2018 [50] South Korea Case report	*N* = 1 Female Age: 30 y	Kerosene (lamp oil) Ingestion Residential	Self reported, no measurement conducted	No predefined outcome assessed Clinical evaluation, diagnostic tests	N/A	N/A	Shortness of breath, cough, fever, right pleuritic pain, decreased breath sounds with inspiratory rales and crackle on right side, right pleural effusion, dyspnea Diagnostic tests: right middle lobe/right lower lobe consolidation with right pleural effusion, necrotic consolidation, ground glass opacity, bronchial wall thickening in right middle/lower lobe and right pleural effusion, edematous and erythematous changes in right middle and lower lobe with grayish secretions on bronchoscopy, slightly turbid, multiple pneumatoceles in right middle lobe Diagnosis: kerosene induced pneumonitis
Long & Charles, 2018 [51] NR (author affiliation: U.S.) Case report	*N* = 1 Male Age: 22 y	Jet Fuel (JP-5) Dermal, inhalation Occupational (Military)	Self reported, no measurement conducted	No predefined outcome assessed Clinical evaluation, audiometric testing	N/A	N/A	Imbalanced, dizzy, difficulty walking, severe left ear pin, thin yellow drainage from ear, muffled hearing, sensation of ear pressure, desquamation and wet-appearing skin with debris, tympanic membrane had dull whitish-appearing excoriations with erythema. Diagnosis: acute noninfectious otitis externa of the left ear, secondary to chemical/fuel exposure
Miko et al., 2023 [2] U.S. Cross-sectional	Survey (*N* = 2,289): Female (59%) Male (41%) Age: 0–84 y HPC (*N* = 147): Female (57%) Male (35%) Age: 0–69 y ED (*N* = 22): Sex and Age not disclosed	Jet Fuel (JP-5) Ingestion, dermal, inhalation Residential, occupational (Military and non-military), community	Self reported, no measurement conducted	New and worsening symptoms, and duration of symptoms Self-reported data, clinical data, electronic health record data	No statistical models stated None, proportions	None	Adults & children: headache, dry/itchy skin, sleepiness/fatigue, diarrhea, dizziness, anxiety, eye/irritation/burning, nausea, difficulty sleeping, skin irritation/burning, skin rash, difficulty concentrating, agitation/irritability, tension/nervousness, difficulty remembering, burning nose/throat, runny nose, feeling depressed, increased tearing, confusion, ringing in ears, difficulty breathing/shortness of breath, coughing, chest tightness or pain/angina, vomiting, paranoia, wheezing, burning lungs, nose bleeds, skin blisters, loss of consciousness/fainting, seizures/convulsions, sore throat
Oba-Daini et al., 2020 [52] Nigeria Cross-sectional	*N* = 31 Female (32%) Male (68%) Age: 7 m − 14 y	Kerosene Ingestion Residential, community	Self reported, no measurement conducted	Clinical features and outcomes Hospital records	None, proportions	N/A	Dyspnea, fever, cough, vomiting, abdominal pain
Oreh et al., 2023 [53] Nigeria Case series	*N* = 4 Female (25%) Male (75%) Age: 15, 24, 24, 36 m	Kerosene Ingestion Residential, community	Self reported, no measurement conducted	No predefined outcome assessed Clinical evaluation, diagnostic tests	N/A	N/A	Cough, vomiting, restless, febrile with fine crepitations heard in middle and lower lung zones bilaterally, noisy breathing, tachypneic, rapid breathing, vomiting, intermittent cough, excessive crying, low-grade fever Diagnoses (all patients): chemical pneumonitis secondary to kerosene poisoning
Parekh & Gupta, 2017 [54] India Cross-sectional	*N* = 42 Female (29%) Male (71%) Age: 0–3 y	Kerosene Ingestion Residential	Self reported, no measurement conducted	Epidemio-toxicological profile of kerosene poisoning Retrospective analysis of medical records	None Proportions	None	Fever, cough, tachypnoea, vomiting, pneumonia, central nervous system symptoms (drowsiness, restlessness, stupor and convulsions). Those with pneumonia had increased bronchiovascular markings and pneumonitis. Mortality due to aspiration pneumonia.
Poon et al., 2019 [55] NR (author affiliation: U.S.) Case report	*N* = 1 Male Age: 33 y	Jet Fuel (JP-8) Inhalation Occupational (Military)	Self reported, no measurement conducted	No predefined outcome assessed Clinical evaluations, diagnostic tests	N/A	N/A	Sudden, sharp right sided and pleuritic chest pain, dry cough, progressive exertional dyspnea, decline in exercise tolerance Diagnostic tests: decreased breath sounds and right sided pneumothorax
Radhakrishnan et al., 2017 [56] India Cross-sectional	*N* = 109 Male (100%) Age: 21–53 y	Aviation fuel (ATF K-50) Dermal Occupational (Military)	No measurement conducted, occupational role used to establish exposure	Cutaneous adverse effects/dermatological manifestations Detailed history, dermatological examination, occlusive patch tests (*N* = 5), potassium hydroxide (KOH) mount	None Proportions	None	Irritant contact dermatitis: itching, transient whitening of skin, rash, scaling Personal history of atopy predisposes personnel to irritant effects from ATF/lubricants.
Ravikanth et al., 2018 [57] NR (author affiliation: India) Case report	*N* = 1 Male Age: 70 y	Kerosene Inhalation NR	Self reported, no measurement conducted	No predefined outcome assessed Clinical evaluation, diagnostic tests	N/A	N/A	Fever, cough with expectoration, breathlessness, signs of tachycardia, tachypnea, diminished breath sounds, crepitations Diagnostic tests: volume loss with air-fluid level in retrocardiac region on right side, costophrenic angle blunting with thickening of right minor fissure, fistulous track between right lower bronchus and pleural cavity, pleural collection along right posterior chest wall with air-fluid level and posterior basal subsegmental bronchus leading into the collection, surrounding subsegmental regional consolidation with mild contralateral mediastinal shift Diagnosis: Bronchopleural fistula due to chemical pneumonitis by aspiration of kerosene
Reddy et al., 2020 [58] India Case series	*N* = 23 Female: 10 Male: 13 Age: 1–5 y	Kerosene (Liquid mosquito repellent) Ingestion NR	Self reported, no measurement conducted	Clinical manifestations of poisoning Hospital records	Univariate and multivariate regression None, frequencies, proportions, means	N/A	Rapid breathing, vomiting, altered sensorium (irritability, lethargy, drowsiness), fever, seizures, mortality (following respiratory distress, altered sensorium, and cardiopulmonary arrest) Clinical tests: bilateral infiltrates, right infiltrates, acute respiratory distress syndrome, chest retractions, crepitations, bronchial breathing, wheeze
Salam et al., 2020 [59] U.S. Case report	*N* = 1 Male Age: 50 y	Jet Fuel Inhalation, dermal Occupational (Aviation)	Self reported, no measurement conducted	No predefined outcome assessed Clinical evaluation, diagnostic tests	N/A	N/A	Epigastric pain, nausea, episodes of nonbilious, non-bloody emesis, confusion, subjective fevers, chills, cough, decline in appetite/oral intake, oliguric and in acute renal failure Diagnostic tests: positive Hepatitis B, mildly echogenic kidneys consistent with medical renal disease
Sanju et al., 2020 [60] India Case report	*N* = 1 Male Age: 6 y	Kerosene Ingestion Not stated	Self reported, no measurement conducted	No predefined outcome assessed Clinical evaluation, diagnostic tests	N/A	N/A	Vomiting, severe abdominal pain, epigastric tenderness Diagnostic tests: pancreatitis, pleural effusion
Subramanian et al., 2018 [61] India Hospital based descriptive study	*N* = 43 Female (36%) Male (64%) Age: 1 m − 12 y^†^	Kerosene Ingestion Residential, community	Self reported, no measurement conducted	Adverse health effects at time of presentation Hospital records	None Proportions	N/A	Pneumonitis, aspiration pneumonia

*ABR* Automatic Neuropsychological Assessment Metrics Version 4, *DPOAE* Distortion product otoacoustic emissions, *ED* Emergency department, *HPC* Hawaiʻi Poison Control, *JP-5* Jet Propellant, *JP-8* Jet Propellant, *mAIAD* modified Amsterdam Inventory for Auditory Disability, *N/A* Not applicable, *NR* Not reported, *PANAS* Positive and Negative Affect Scale, *THC* total hydrocarbons, *U.S.* United States

* Indicates analytical studies

† Age and sex generalized across all poisonous agents, numbers specific to kerosene poisoning not provided

### Quality assessment and risk of bias

To evaluate study rigor, a critical appraisal was performed [31, 62]. This entailed a quality assessment of all included publications and an OHAT risk of bias assessment on epidemiological studies assessing associations between exposure and outcomes of interest to this review. Quality assessment evaluates how well individual studies were designed, conducted and reported. Four distinct checklists were applied to determine quality assessment scores of included studies according to their design: trials, case-control/cohort studies, cross-sectional studies and case reports/case series, and can be found in Additional files 4–6. These checklists were adapted from the Modified Downs and Black Checklist (trials, case-control/cohort, and cross-sectional) and the Joanna Briggs Checklist for Case Reports (case reports and case series) [63, 64]. Because these checklists were not created for studies in environmental health, additional scoring items evaluating exposure assessment were added for appraisals of trials, case-control/cohort and cross-sectional studies based on a recent systematic review in environmental epidemiology [65]. The full appraisal tools, including items assessed and corresponding scoring criteria are provided in Additional files 4–6. To maintain consistency and transparency, each study was evaluated using the appraisal framework corresponding to its self-identified design. One study did not self-identify its design [52]; given that their methods mirrored other descriptive cross-sectional studies identified in this review, the article was quality assessed against that framework. Quality assessment was conducted blindly and independently by two reviewers (VC and BN). Consensus between reviewers was required to finalize quality assessment scores. Total scores consisted of the combined scores of all indicators, which were then expressed as a percentage of the maximum score. Categories of quality were assigned based on the score in percentage: excellent quality (score ≥ 81%), good quality (between 61 and 80%), fair quality (between 41 and 60%), poor quality (between 21 and 40%) and very poor quality (≤ 20%).

The OHAT risk of bias (RoB) assessment was conducted to evaluate the internal validity of included epidemiological studies reporting on associations. Internal validity is whether the study is free from systematic error, or bias, and is assessed to determine whether the reported associations are likely to be “true” [66]. Two researchers (VC and NR) independently performed RoB appraisals at the study level, determining risk of bias within six bias domains as either “definitely low”, “probably low”, “probably high” or “definitely high” according to OHAT criteria [31]. The bias domains assessed included selection, confounding, attrition/exclusion, detection, selective reporting and other sources of bias [31]. The researchers met to compare results and a third reviewer (CP) was consulted for resolution of one disagreement in determination. As case-reports and case-series are descriptive reports and do not estimate associations, these articles were not subject to a RoB assessment.

### Analysis & evaluation of evidence

Observable and/or diagnosable health outcomes were prioritized, including symptoms, conditions, clinical signs/observations, and performance-based outcome measures. Laboratory and physiological clinical measurements (e.g. blood count, respiratory rate, heart rate, oxygen saturation (SpO_2_)) were excluded from analyses given the absence of patient baseline values limited meaningful interpretation of these results. Diagnostic imaging findings (e.g. X-ray, computed tomography, magnetic resonance imaging) which reflect structural and anatomical aberrations were retained for analysis.

Health outcomes were extracted verbatim as reported in the included studies and searched for within the International Classification of Diseases, 10th revision (ICD-10) to confirm appropriate grouping by bodily system [67]. When the reported health outcomes did not yield a direct match in the ICD-10, corresponding clinical terms were identified and applied to assist in the search. When needed, the context of the included study was used for classification. To facilitate synthesis and analysis, all reported symptoms were further categorized within each bodily system. For example, individual symptoms and diagnoses were organized into symptom categories (e.g. “dyspnea”, “shortness of breath” and “rapid breathing” were grouped under the category “Laboured breathing” within the Respiratory system).

Binary coding was conducted at the study level. For each study, a value of 1 assigned when authors reported at least one symptom in the corresponding category. Thus, frequency counts reflect the number of studies reporting a given symptom category, rather than the number of affected individuals. These counts were used to assess the distribution and consistency of health outcomes across studies, and stratified by exposure type (kerosene or jet fuel), and route (oral, dermal, or inhalation) when possible. Full mappings of extracted health outcomes and categories can be found in Additional file 7.

The quality assessment and OHAT risk of bias scores account for design and methodological limitations of a given study, and were used to contextualize the evidence in our synthesis and evaluation of reported findings. A subset analysis limited to good and excellent quality studies was conducted where frequency counts were reassessed to evaluate health outcomes supported by higher quality evidence. These patterns were then compared to those observed across all included studies to evaluate consistency of findings. For results derived from epidemiological studies estimating associations between kerosene or jet fuel and health outcomes, the risk of bias scores were used to further inform interpretation of findings.

## Results

### Selected studies

A total of 2,216 articles were retrieved from the search. After deduplication, and title and abstract screening, 72 articles remained for full text review. One additional eligible article was found upon retrospective citation tracing. Ultimately, 28 articles met all eligibility criteria and were included in this review. The PRISMA flow diagram outlining the article selection process is presented in Fig. 1.

### Characteristics of included studies

#### Study design

All included studies were observational in design; no randomized controlled trials or other intervention studies were identified. Three studies (11%) were analytical based on their use of comparison groups to assess relationships between exposure to JP-5 or JP-8 and human health outcomes: two cross-sectional studies [41, 44] and one cohort study [49]. The vast majority of included studies (89%) were descriptive in nature and did not estimate exposure-outcome associations, including case reports (*N* = 14), cross-sectional studies (*N* = 6), case series (*N* = 3), one “hospitable based descriptive study” and one ethnographic study.

#### Participant demographics

Males were represented in 23 (85%) studies, compared to females who were assessed in 15 (56%) and comprised at least 50% of the sample in only 6 (22%) articles. The age group most observed across included articles were adults of working age (aged 18 to 64 years) (*N* = 14), followed by children (aged 2 to 17 years) (*N* = 12), infants (up to and including one year) (*N* = 7) and older adults (aged 65 and older) (*N* = 4). However, in kerosene-specific studies, children (*N* = 10, 59%) and infants (*N* = 6, 35%) were most assessed, compared to those specific to jet fuel in which the vast majority of reports assessed adults of working age (*N* = 10, 91%). Sample sizes ranged from single-patient case reports to a survey of 2,289 participants [2]. A summary of participant age groups by fuel type is presented in Table 3.

**Table 3:**
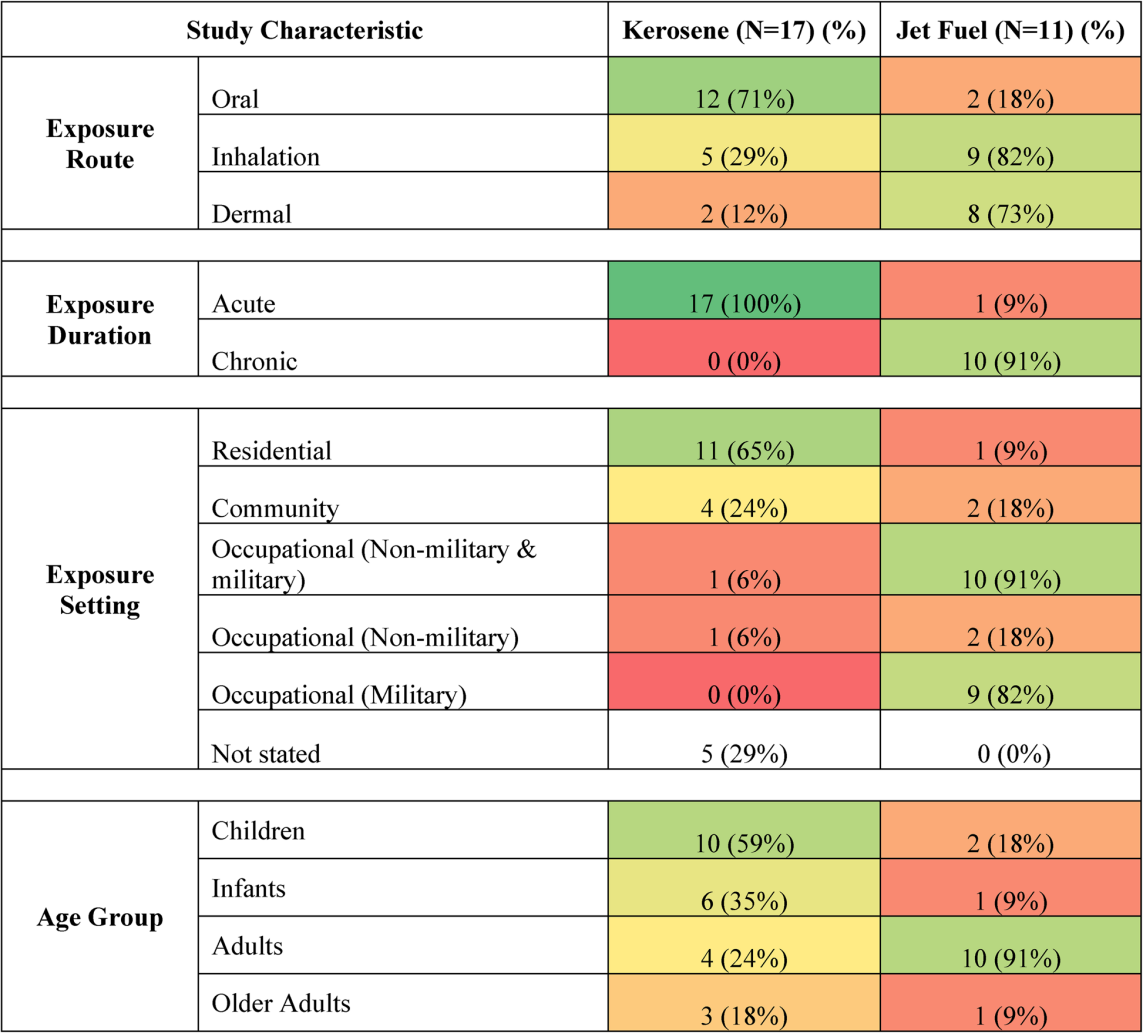
Characteristics of Included Studies. This frequency heat table compares key characteristics of included studies, including exposure context and age of participants, stratified by fuel type

*Number (N) and proportion (%) of studies reporting characteristic. Cell shading used to visually represent frequency distributions, with green indicating more reports and red indicating less

#### Country and year of publications

Kerosene-specific reports appeared consistently throughout the review period (2017–2024), with the exception of 2019, and were published primarily in LMIC, including India (*N* = 8) and Sri Lanka (*N* = 1). Other kerosene-specific reports originated from Japan (*N* = 2), South Korea, Spain and the U.S. (*N* = 1 each). In contrast, most jet fuel-specific articles originated from higher-income countries, including the U.S. (*N* = 8) and Australia (*N* = 1) with the remaining studies conducted in Kenya and Uganda (*N* = 1) and in India (*N* = 1). Only 3 (27%) jet fuel-specific studies were published after 2020, with none identified in the years 2021 or 2024.

### Quality assessment and risk of bias appraisal

Average quality scores varied by study design, with case reports scoring highest on average at 77% (“good” quality). These publications typically scored well on items regarding descriptions of patients and their presenting conditions, but scored poorly when assessed for reporting of unanticipated or harmful events following clinical treatment. Cross-sectional studies were generally of “fair” quality with an average score of 49%. Common limitations of these studies included insufficient, or lack of: reporting on participant recruitment and demographic characteristics, consideration of confounders, formal exposure assessment, as well as analyses needed to draw dose-response conclusions about the associations of interest for this review. The sole cohort study identified in this review ranked “good” quality with a score of 71% [49]. Notably, two of the three analytical studies identified by this review scored of higher quality (good or excellent) [44, 49]. Only one analytical study scored of poor quality. A summary of quality assessment results can be found in Table 4. Due to the absence of comparable studies, quality assessment was not conducted on one descriptive ethnographic study [36], one “hospital based descriptive study” [61] and one analytical case series [58]. The scores for each quality assessment item for all included studies are presented in Additional files 8–10.

**Table 4:** Results of quality assessment. This table summarizes quality assessment results of each included study, specifying study design, score in percent and corresponding quality category

Author & Year	Study Design	Score (%)	Score Quality Category
Kim et al., 2018 [50]	Case report	90	Excellent
Long & Charles, 2018 [51]	Case report	90	Excellent
Poon et al., 2019 [55]	Case report	90	Excellent
Benítez Riesco et al., 2024 [37]	Case report	70	Good
Chattopadhyay et al., 2021 [38]	Case report	80	Good
Contestable, 2017 [39]	Case report	80	Good
Esashi et al., 2021 [42]	Case report	70	Good
Fuente et al., 2019 [44]	Cross-sectional	71	Good
Goenka et al., 2022 [45]	Case report	80	Good
Gupta et al., 2017 [46]	Case report	70	Good
Guss et al., 2020 [47]	Case report	70	Good
Hara et al., 2018 [48]	Case report	80	Good
Heaton et al., 2017 [49]	Cohort	70	Good
Oreh et al., 2023 [53]	Case report	80	Good
Ravikanth et al., 2018 [57]	Case report	80	Good
Salam et al., 2020 [59]	Case report	70	Good
Sanju et al., 2020 [60]	Case report	80	Good
Areprekumor et al., 2024 [35]	Cross-sectional	43	Fair
Dayasiri et al., 2017 [40]	Cross-sectional	48	Fair
Dreisbach et al., 2022 [41]	Cross-sectional	52	Fair
Fife et al., 2018 [43]	Case report	50	Fair
Miko et al., 2023 [2]	Cross-sectional	48	Fair
Parekh & Gupta, 2017 [54]	Cross-sectional	48	Fair
Radhakrishnan et al., 2017 [56]	Cross-sectional	48	Fair
Oba-Daini et al., 2020 [52]	Cross-sectional	38	Poor
Bateganya & Nakanjako, 2023* [36]	Ethnographic study	N/A	N/A
Reddy et al., 2020* [58]	Analytical case series	N/A	N/A
Subramanian et al., 2018* [61]	Hospital-based descriptive study	N/A	N/A

* Quality assessment not conducted due to lack of comparison studies

Three included studies were eligible to undergo a risk of bias appraisal; one cohort [49], and two cross-sectional studies [41, 44]. Results of these evaluations generally revealed a lower risk of bias in areas of data attrition/exclusion, outcome assessment, and selective reporting. Studies typically demonstrated a higher risk of bias in confounder consideration and exposure characterization. No study was determined to demonstrate a consistently high or low risk of bias across all domains. Detailed scores for each study can be found in Additional file 11.

### Characteristics of exposure

A summary of exposure durations, settings and routes by fuel type is presented in Table 3.

#### Exposures and durations assessed

Jet fuel-specific studies comprised 39% of included publications (*N* = 11) [2, 36, 39, 41, 43, 44, 49, 51, 55, 56, 59]. Among these, JP-5 (*N* = 4) and JP-8 (*N* = 4) were most frequently assessed. Other less reported fuels included JP-4 or generalized jet/aviation fuel without specification of subtypes. Of these 11 jet fuel specific-studies, six (55%) scored as good or excellent quality [39, 44, 49, 51, 55, 59]. The greater part of all jet-fuel specific studies involved chronic occupational exposures (*N* = 10, 91%).

Kerosene-specific reports comprised the larger portion of included studies (*N* = 17, 61%), all of which involved acute exposure events [35, 37, 38, 40, 42, 45–48, 50, 52–54, 57, 58, 60, 61]. Five of these studies reported on health outcomes of kerosene-containing products, including lamp oil, sailboat lubricant, wood preservative and Indigenous pesticides, while the remaining articles assessed pure kerosene specifically. Eleven (65%) of these kerosene-specific reports ranked of higher quality [37, 38, 42, 45–48, 50, 53, 57, 60].

#### Exposure setting

Exposure in residential (*N* = 12) and occupational (*N* = 11) settings were most frequently reported across studies; however, exposure contexts varied widely by fuel type. Of the kerosene-specific studies that reported an exposure setting, all but one reported instances occurring in the home (*N* = 11); only one study reported residential exposure to jet fuel [2]. Nearly all jet fuel-specific studies assessed occupational exposure (*N* = 10), predominantly in military (*N* = 9) compared to non-military settings (*N* = 2). Only one study reported workplace exposure to kerosene (*N* = 1). Exposures occurring in the community were less frequently reported for kerosene (*N* = 4) and jet fuel (*N* = 2).

#### Exposure routes

Patients and participants were most exposed through ingestion (*N* = 14) and inhalation (*N* = 14) across all included studies. Dermal exposures were also commonly reported (*N* = 10). When stratified by fuel type, the predominant routes of exposure varied widely; studies assessing kerosene most frequently involved ingestion (*N* = 12, 71%), with fewer documenting inhalation (*N* = 5, 29%) or dermal (*N* = 2, 12%) routes. Jet fuel was primarily experienced through inhalation (*N* = 9, 82%) and dermal (*N* = 8, 73%) routes. Notably, oral exposure to jet fuel was identified in only two publications (18%) [2, 44].

#### Exposure assessment methods

Exposure was most frequently established through self-reported data (*N* = 24, 86%). This was especially common in case reports, case series, and cross-sectional studies which made up the majority of included studies. All kerosene-specific publications, and many jet fuel studies (*N* = 7, 64%), relied on patient or caregiver-reported exposure documented during clinical evaluations. Only three studies, all of which assessed jet fuel, conducted formal exposure assessments: Two analytical studies sampled personal air [41, 49], and urine [49]. In the third study, proxies including task group and job history, self-reported exposure level, and previous industrial assessments, were utilized to estimate exposures [44]. One descriptive cross-sectional study identified occupational role (e.g. aircraft ground crew) as a proxy to presume exposure to aviation turbine fuel and lubricants, but did not assess exposure levels or compare outcomes across exposure gradients [56].

### Health outcomes by bodily system and fuel type

A detailed overview of frequency counts for individual symptom categories within each bodily system, by fuel type and study quality, is provided in Tables 5 and 6.

**Table 5:** Health effects reported across kerosene-specific studies. This table summarizes health effects by bodily system and symptom categories for kerosene-specific studies, stratified by quality

Health Effect	Overall (*N* = 17) (%)	Good/Excellent Quality (*N* = 11) (%)
*Respiratory*	14 (82%)	8 (73%)
Laboured Breathing	10 (59%)	6 (55%)
Infectious/Inflammatory Lung Conditions	9 (53%)	6 (55%)
Cough	9 (53%)	5 (45%)
Tissue/Mechanical Impacts	8 (47%)	7 (64%)
Abnormal Breath Sounds	7 (41%)	6 (55%)
Chest Discomfort	1 (6%)	1 (9%)
Other Respiratory Impacts	1 (6%)	0
Upper Respiratory Irritation	0	0
*Gastrointestinal*	*7* (41%)	*2* (18%)
Vomiting	7 (41%)	2 (18%)
Abdominal Pain	3 (18%)	1 (9%)
Nausea	1 (6%)	0
Other	2 (12%)	2 (18%)
Appetite Decline	0	0
Diarrhea	0	0
*Neurological*	*6* (35%)	*2* (18%)
Impaired Consciousness	5 (29%)	1 (9%)
Cognitive Function & Processing	2 (12%)	1 (9%)
Seizures & Convulsions	2 (12%)	0
Equilibrium & Spatial Disorientation	1 (6%)	0
Mood & Affective Symptoms	1 (6%)	0
Auditory Impacts	0	0
Head Pain & Discomfort	0	0
Ocular Symptoms	0	0
Sensory Impairment	0	0
*Fever*	*5* (29%)	*1* (9%)
*Mortality*	*4* (24%)	*0*
*Dermatological*	*3* (18%)	*3* (27%)
Contact Dermatitis/Irritation	2 (12%)	2 (18%)
Changes to Skin Barrier	2 (12%)	2 (18%)
Features of Infection/Abscess	2 (12%)	2 (18%)
*Cardiovascular*	*2* (12%)	*0*
*Methaemoglobinaemia*	*1* (6%)	*1* (9%)

**Table 6:** Health effects reported across jet fuel-specific studies. This table summarizes health effects by bodily system and symptom categories for jet fuel-specific studies, stratified by quality

Health Effect	Overall (*N* = 11) (%)	Good/Excellent Quality (*N* = 6) (%)
*Neurological*	*8* (73%)	*4* (67%)
Auditory Impacts	5 (45%)	2 (33%)
Cognitive Function & Processing	5 (45%)	3 (50%)
Equilibrium & Spatial Disorientation	3 (27%)	1 (17%)
Head Pain & Discomfort	2 (18%)	0
Impaired Consciousness	2 (18%)	0
Mood & Affective symptoms	2 (18%)	0
Ocular Symptoms	2 (18%)	0
Sensory Impairment	2 (18%)	1 (17%)
Seizures & Convulsions	1 (9%)	0
*Dermatological*	*5* (45%)	*2* (33%)
Contact Dermatitis/Irritation	5 (45%)	2 (33%)
Changes to Skin Barrier	2 (18%)	1 (17%)
Features of Infection/Abscess	0	0
*Respiratory*	*4* (36%)	*2* (33%)
Cough	4 (36%)	2 (33%)
Chest Discomfort	3 (27%)	1 (17%)
Laboured Breathing	3 (27%)	1 (17%)
Upper Respiratory Irritation	2 (18%)	0
Other Respiratory Impacts	1 (9%)	1 (17%)
Abnormal Breath Sounds	1 (9%)	1 (17%)
Infectious/Inflammatory Lung Conditions	0	0
Tissue/Mechanical Impacts	0	0
*Gastrointestinal*	*3 (27%)*	*1* (17%)
Abdominal Pain	2 (18%)	1 (17%)
Appetite Decline	2 (18%)	1 (17%)
Nausea	2 (18%)	1 (17%)
Vomiting	2 (18%)	1 (17%)
Diarrhea	1 (9%)	0
Other	0	0
*Renal*	*2* (18%)	*1* (17%)
*Ocular*	*2* (18%)	*0*
*Cardiovascular*	*1* (9%)	*0*
*Mortality*	*1* (9%)	*0*
*Fever*	*1* (9%)	*1* (17%)

#### Respiratory

Impacts to the respiratory system were most observed, reported in 18 descriptive studies (64%), following oral (*N* = 13), inhalation (*N* = 9) and dermal (*N* = 3) exposures which often co-occurred. The most frequently documented respiratory symptoms following kerosene and jet fuel exposures included laboured breathing (e.g. breathlessness, dyspnea and tachypnea) (*N* = 13) [2, 40, 42, 43, 45, 48, 50, 52–55, 57, 58] and cough (*N* = 13) [2, 35, 40, 42, 43, 45, 50, 52–55, 57, 59], followed closely by infectious or inflammatory lung conditions (*N* = 9), tissue or mechanical impacts (*N* = 8) and abnormal breath sounds (*N* = 8). Less frequently reported symptoms included chest discomfort, upper respiratory irritation and other respiratory impacts (i.e. respiratory distress and decline in exercise tolerance).

When restricting analyses to good or excellent quality studies, respiratory symptoms remained the most frequently reported health outcomes (*N* = 10) [38, 42, 45, 48, 50, 53, 55, 57, 59, 60]. Similar symptom patterns were observed in this subset: laboured breathing, tissue or mechanical impacts, abnormal breath sounds and cough were most frequently identified (*N* = 7, each), followed closely by infectious or inflammatory lung conditions (*N* = 6). Upper respiratory irritation, however, did not appear in studies of higher quality.

##### Respiratory - kerosene

Respiratory outcomes were recorded in the greater part of all kerosene-specific studies (82%, *N* = 14) [35, 38, 40, 42, 45, 48, 50, 52–54, 57, 58, 60, 61]. Most respiratory complaints involved oral exposures (*N* = 12), with fewer documenting inhalation (*N* = 5). Laboured breathing (*N* = 10) [40, 42, 45, 48, 50, 52–54, 57, 58] and infectious or inflammatory lung conditions, including aspiration pneumonia and chemical pneumonitis (*N* = 9) [35, 38, 42, 45, 48, 50, 54, 57, 61], appeared most in kerosene-specific studies. Cough (*N* = 9), tissue or mechanical impacts (*N* = 8), as well as abnormal breath sounds (*N* = 7) were also commonly noted; whereas, chest discomfort and general respiratory distress appeared less. Upper respiratory irritation was not reported following kerosene exposure, and no respiratory symptoms were observed with dermal contact.

Among the 13 studies reporting health outcomes from kerosene exposure, eight were of good or excellent quality [38, 42, 45, 48, 50, 53, 57, 60]. Tissue or mechanical impacts such as decreased air entry and emphysematous changes were the most prevalent respiratory impacts (*N* = 7) within these studies, followed closely by laboured breathing, infectious or inflammatory lung conditions, abnormal breath sounds (*N* = 6, each), and cough (*N* = 5). Chest discomfort, while less prevalent, was also supported by higher quality evidence.

##### Respiratory - jet fuel

Among 11 jet-fuel specific studies, respiratory outcomes were documented in four (36%), all of which involved inhalation of JP-4, JP-5, or JP-8 specifically [2, 43, 55, 59]; however, dermal (*N* = 3) and oral (*N* = 1) routes often occurred simultaneously. Cough was the most consistent respiratory complaint, reported in each of the four studies [2, 43, 55, 59]. Symptoms of chest discomfort such as angina, chest pressure and pleuritic pain [2, 43, 55], and laboured breathing [2, 43, 55] were also detected. Upper respiratory irritation and abnormal breath sounds were less prevalent among these studies, with one documenting a decline in exercise tolerance.

Respiratory symptoms following jet fuel exposure were supported by two studies of higher quality [55, 59] with reports of cough consistent across both. Chest discomfort, laboured breathing, abnormal breath sounds and other respiratory impacts were each reported in only one study of good or excellent quality. No higher quality evidence suggested infectious or inflammatory lung conditions, tissue or mechanical impacts, or upper respiratory irritation with jet fuel exposure.

#### Neurological and auditory

Evidence of neurological and/or auditory impacts were reported in 14 studies (50%) [2, 35, 36, 40–44, 48, 49, 51, 54, 58, 59] following inhalation (*N* = 12), oral (*N* = 7), and dermal (*N* = 6) exposures to kerosene and/or jet fuel. Impaired consciousness [2, 35, 40, 43, 48, 54, 58], and impacts on cognitive function and processing [2, 42–44, 49, 54, 59] were the most consistent neurological outcomes overall (*N* = 7, each). These symptoms were followed closely by auditory impacts (*N* = 6) [2, 41, 43, 44, 49, 51].

When limited to studies of good or excellent quality, neurological outcomes remained the second most frequently reported across included studies (*N* = 6) [42, 44, 48, 49, 51, 59]. Higher quality evidence supported impacts on cognitive function and processing to be the most consistent neurological symptoms (*N* = 4). Auditory impacts, equilibrium and spatial disorientation, impaired consciousness and sensory impairment were less frequently observed among these reports. Other symptoms observed in lower quality studies such as head pain and discomfort, mood, affective, or ocular symptoms, or seizures and convulsions, were not supported by findings of good or excellent quality reports.

##### Neurological and auditory - kerosene

Neurological impacts were recorded in 35% of studies reporting on kerosene (*N* = 6) [35, 40, 42, 48, 54, 58], largely connected to oral (*N* = 5) and inhalation (*N* = 4) exposures. All evidence of neurological health outcomes following kerosene exposure were derived from descriptive studies. Although impaired consciousness (e.g. delirium, drowsiness and fainting) was most identified (*N* = 5) [35, 40, 48, 54, 58], only one report ranked above fair quality [48].

Two studies of good or excellent quality noted neurological outcomes of kerosene exposure [42, 48], specifically impaired consciousness and impacts on cognitive function and processing. Other symptoms including seizures and convulsions, mood and affective impacts as well as equilibrium and spatial disorientation appeared less and were not supported by higher quality evidence. No studies, regardless of quality, observed auditory impacts or sensory impairment following kerosene exposure.

##### Neurological and auditory - jet fuel

Neurological and/or auditory symptoms following jet fuel were identified in eight publications, including both descriptive reports and analytical epidemiological studies (73%) [2, 36, 41, 43, 44, 49, 51, 59]. While all instances involved inhalation exposure, dermal (*N* = 6) and oral (*N* = 2) exposures were often co-occurring.

Four studies were rated of good or excellent quality [44, 49, 51, 59]. Impaired cognitive function and processing (including visual memory impacts, confusion, and difficulty concentrating), were among the most widely reported neurological symptoms overall [2, 43, 44, 49, 59], and in higher quality studies [44, 49, 59]. While indicators of equilibrium and spatial disorientation such as dizziness, stumbling, and sensory impairment (e.g. loss of pain, cold and hunger sensations) were reported less, these symptoms also appeared in at least one higher quality study. In contrast, head pain and discomfort, impaired consciousness, mood, affective and ocular symptoms, as well as seizures and convulsions were documented less overall and only in poorer quality studies.

Two analytical studies tested associations between jet fuel and neurological outcomes. Both were of high quality and observed higher JP-8 inhalation to be associated with poorer cognitive function and processing among male and female adults of working age [44, 49]. Fuente et al. reported a dose-response relationship as increased inhalation, dermal and oral exposure resulted in poorer central auditory processing and compressed speech among members of the Royal Australian Air Force (2019) [44]. These results however, are subject to potentially high confounding and exposure characterization bias, as identified during the OHAT RoB appraisal. Similarly, greater JP-8 inhalation in a cohort of U.S. Air Force personnel was associated with decreased performances in visual memory and motor speed, though fine motor performance (measured through Grooved Pegboard Test) improved among those more highly exposed [49]. This study also had limitations pertaining to inadequate consideration of confounders and other sources of bias.

Auditory impacts following jet fuel exposure were documented in five studies (45%), most of which were fair quality [2, 41, 43], with only two scoring good or excellent [44, 51]. In one case report of excellent quality, a military aviation technician of 22 years of age complained of severe ear pain, drainage, muffled hearing and a sensation of ear pressure following an incident in which JP-5 entered his ear canal after splashing his face [51]. Audiometric testing, however, confirmed the patient’s hearing was within normal limits [51]. These findings contrast with those of Fuente et al. who, in an analytical cross-sectional study of good quality, observed higher JP-8 exposures among Royal Australian Air Force personnel to be associated with significantly worse hearing thresholds (at 4kHZ in both ears, and 8 kHz in the right ear) and poorer average hearing thresholds across all frequencies (1–8 kHz) in participant’s “better” ear (2019) [44]. Additionally, increased exposure was associated with significantly worse results on pure-tone thresholds, distortion product otoacoustic emissions, auditory brainstem response wave V latency, and scores for words-in-noise [44]. With these results, Fuente et al. suggest that jet fuel, when combined with noise exposure, adversely impacts the human auditory system (2019) [44]. These results should be interpreted with the same consideration of risk of confounding and exposure characterization biases noted previously for this study. Similar symptoms were reported among descriptive studies of poorer quality, including increased difficulty in listening situations, ear pain, hearing loss, and ringing in ears, following oral, dermal and inhalation exposures to jet fuel. An analytical study reported worse speech intelligibility in noise among participants exposed to JP-5 compared to those unexposed [41]. These results are constrained by concerns of lower study quality and a high risk of bias across several OHAT domains, most notably in participant selection, confounding consideration and exposure assessment.

#### Gastrointestinal

Reports of gastrointestinal outcomes were present in eleven descriptive reports (36%) [2, 35, 36, 40, 42, 52–54, 58–60], with vomiting being the most prevalent symptom overall (*N* = 8) [2, 35, 40, 53, 54, 58–60]. Gastrointestinal symptoms often stemmed from oral (*N* = 9) or inhalation (*N* = 6) exposures, with dermal contact (*N* = 2) noted less among these articles.

Among good or excellent quality studies reporting gastrointestinal outcomes, vomiting was consistently documented [53, 59, 60]. Although abdominal pain, decline in appetite, nausea and pancreatitis appeared less overall, these symptoms were supported by at least one higher quality study. Diarrhea was reported exclusively in one poorer quality report [2].

##### Gastrointestinal - kerosene

Gastrointestinal outcomes were observed in eight kerosene-specific studies (41%) [35, 40, 42, 52–54, 58, 60] often following ingestion (*N* = 8); whereas, inhalation was noted in only three of these reports [35, 40, 42]. Vomiting was consistently reported in these studies, two of which were higher quality [53, 60]. Abdominal pain and pancreatitis were also observed in one report of higher quality [60], while nausea was documented in one lower quality study [40].

##### Gastrointestinal - jet fuel

Gastrointestinal symptoms were recorded in 27% (*N* = 3) of jet fuel-specific studies [2, 36, 59], all of which followed inhalation exposure, often in combination with dermal (*N* = 2) and oral (*N* = 1) routes. While no symptom was recorded consistently across these studies, vomiting, abdominal pain, appetite decline, and nausea appeared most within this subset (*N* = 2 each). These findings were further supported by one high quality case report [59]. Diarrhea was noted in only one lower quality study [2].

#### Dermatological

Dermatological manifestations were documented in 29% of all included studies (*N* = 8) [2, 37, 39, 43, 47, 48, 51, 56], nearly all involving dermal contact (*N* = 7). Inhalation (*N* = 4) and oral (*N* = 1) exposures were noted less among these reports. Signs of contact dermatitis and/or irritation such as dry, itchy or burning skin, were the most frequently reported dermatological symptoms overall (*N* = 7) [2, 37, 39, 43, 47, 51, 56], and among higher quality studies (*N* = 4) [37, 39, 47, 51]. Although less frequently observed, features of infection or abscess such as ulcers, purulence and phlegmonous changes, as well as skin barrier changes (e.g. desquamation, whitening of skin) were evident in both higher and lower quality studies. Dermal impacts were not assessed within analytical studies.

##### Dermatological - kerosene

Three case reports, all of good quality, reported dermatological symptoms (18%) following dermal [37, 47] and inhalation [48] exposures to kerosene. Contact dermatitis and/or irritation [37, 47], changes to the skin barrier [37, 48] and features of infection and/or abscess [37, 47] were equally reported following kerosene exposure. Additional evidence of dermatological outcomes following kerosene exposure was not demonstrated among poorer quality studies.

##### Dermatological - jet fuel

Five jet-fuel specific studies (45%) observed dermatological outcomes, and each noted contact dermatitis and/or irritation following dermal exposure [2, 39, 43, 51, 56]. Inhalation (*N* = 3) and oral (*N* = 1) exposures were frequently identified alongside dermal contact. Two studies stemmed from higher quality case reports of male U.S. military servicemembers who experienced dermal [39, 51] and inhalation [51] exposures to JP-5. In one case, a 19 year-old U.S. Navy sailor developed a pruritic rash on his hands and forearms that worsened over 10 days following direct contact with a large volume of JP-5 and was ultimately diagnosed with irritant contact dermatitis [39]. While the reported symptoms seemed to follow an isolated incident, the patient reported routine occupational exposure to JP-5 during storing, transferring and refueling operations [39]. Similarly, a 22 year-old military aviation technician was diagnosed with acute non-infectious otitis externa after JP-5 entered his ear canal [51]. Clinical evaluation revealed erythema, and skin barrier alterations including a wet-appearance with debris consisting of large amounts of gray to whitish desquamating skin [51].

Changes to skin barrier were less observed, and appeared in both higher and lower quality studies. Unlike kerosene, features of infection or abscess following jet fuel exposure were not observed among included studies.

#### Less reported health outcomes

Infrequent findings reported following kerosene and/or jet fuel exposures included mortality, cardiovascular, ocular, renal, and miscellaneous (i.e. fever and methemoglobinemia) outcomes, each reported in fewer than six descriptive studies. Higher-quality studies also identified fever (*N* = 3), methaemoglobinaemia, and renal disease as potential outcomes [45, 46, 59].

##### Mortality

Mortality outcomes following kerosene or jet fuel were documented in five included studies, and exclusively among children [35, 36, 40, 54, 58]. Among the four kerosene-specific studies, death followed ingestion in pediatric patients as young as 9 months old [35, 40, 54, 58], with inhalation documented as a potential co-occurring exposure route in two of these reports [35, 40]. Similar findings were identified in a descriptive ethnographic study where premature death was one of two primary side effects of sniffing aviation fuel among child migrants living on the Uganda-Kenya border [36]. However, no evidence of higher quality indicated mortality as an outcome of kerosene or jet fuel exposures.

##### Cardiovascular

Cardiovascular symptoms including erratic heart beats, tachycardia and cardiopulmonary arrest, were documented in three studies following kerosene [40, 58] and jet fuel [43] exposures. In both kerosene-specific studies, cardiovascular outcomes including cardiopulmonary arrest resulting in death, occurred in children aged 9 months to 12 years following ingestion of kerosene or a kerosene-based liquid mosquito repellent. Other cardiovascular symptoms, including palpitations and erratic heart beats, were observed in two female U.S. military personnel with 3–5 years of JP-8 inhalation in a poorly ventilated workplace [43]. No high quality studies provided evidence supporting cardiovascular effects of kerosene or jet fuel exposure.

##### Ocular

Ocular outcomes appeared in two publications, both involving jet fuel exposures [2, 43]. Symptoms such as eye irritation and burning, often persisting for more than 30 days post exposure, occurred in both children and adults following oral, dermal and inhalation exposure to JP-5 contaminated groundwater [2]. Similar symptoms were identified in two female U.S. military personnel who presented with blurred vision and eye irritation after 3–5 years of occupational JP-8 and JP-4 exposures [43]. Both reports were of lower quality, and no ocular symptoms were present in higher quality studies or those related to kerosene specifically.

##### Renal

Two accounts of renal outcomes were identified, both in adults of working age following chronic dermal and inhalation exposure to jet fuel [43, 59]. One case report of higher quality documented acute renal failure in a 50 year-old male aircraft refueler who experienced four years of daily inhalation and dermal exposure to jet fuel [59]. Renal outcomes of jet fuel were further supported by one lower quality case report which outlined recurrent bladder and urinary tract infections in two U.S. female service members following chronic JP-8 and JP-4 inhalation [43].

##### Other health outcomes

Miscellaneous health outcomes included reports of fever (*N* = 6) and one hematologic diagnosis (*N* = 1).

Incidences of fever predominantly followed ingestion of kerosene, in both children [35, 52, 54, 58], and adults [45]. Only one high quality case report documented fever, where an adult male patient presented with acute renal failure following chronic inhalation and dermal exposure to jet fuel [59].

Hematological outcomes were outlined in only one study, in which a 28 year-old male was diagnosed with methaemoglobinaemia following ingestion of a kerosene-based Indigenous pesticide in India [46]. While no additional hematological effects were identified, laboratory and clinical measurements, including oxygen saturation levels or blood pressure, were not included in this review unless presented as observable symptoms or clinical diagnoses.

Ultimately, evidence of cancer, endocrine, developmental, hepatic, immune, reproductive, musculoskeletal and metabolic outcomes following exposure to pre-combustion forms of kerosene or jet fuel were not identified among included studies.

## Discussion

Based on this review of 28 publications, respiratory and neurological systems appear most frequently affected by kerosene-based fuels. Gastrointestinal and dermatological outcomes were also commonly observed, noted in approximately one third of all included studies. When stratified by fuel type, respiratory outcomes were most consistently reported following kerosene-specific exposures, whereas neurological effects appeared most among jet fuel specific studies. Three analytical studies were identified, all of which assessed and reported neurological and/or auditory health impacts following jet fuel exposure. This review did not observe evidence of cancer, endocrine, developmental, hepatic, immune, reproductive, musculoskeletal or metabolic outcomes following exposure to either fuel type.

### Scientific evidence of health outcomes following exposure

The most consistent evidence across all studies included respiratory, neurological, gastrointestinal and dermatological outcomes following kerosene and jet fuel exposures. These patterns were supported when limiting analyses to higher quality studies, where dermatological outcomes only increased in prevalence among kerosene-specific reports. Analytical studies consistently demonstrated neurological impacts of jet fuel exposure however, findings were typically limited by concerns surrounding exposure assessment methods and incomplete consideration of critical confounders. Although there is clear overlap in the four systems most impacted overall, stratification by fuel type demonstrates differences in relative frequency and presentation of most prominent health outcomes. These variations may be reflective of differences in chemical composition, as well as duration, dosage and routes of exposures.

Respiratory outcomes appeared in the greater part of all kerosene-specific studies (82%), particularly as infectious or inflammatory lung conditions, laboured breathing, tissue or mechanical impacts, abnormal breath sounds and cough. These findings mirror those consolidated by ATSDR, which documented human respiratory outcomes such as pneumonitis, cough, dyspnea, pulmonary edema and lung infiltrates following kerosene ingestion (2017) [14]. Conversely, respiratory outcomes were evident in approximately one third of jet fuel-specific reports (36%), with the most common symptoms being cough, chest discomfort and laboured breathing. For jet fuel studies, neurological outcomes, notably auditory impacts and impaired cognitive function and processing, were most widely reported (73%), whereas neurological outcomes were documented less following kerosene exposures (38%), and manifested primarily as impaired consciousness. Gastrointestinal outcomes such as vomiting, abdominal pain, decline in appetite, nausea and diarrhea, were recorded in roughly one third (36%) of included studies. These symptoms were more often identified within kerosene-specific literature, which may reflect the frequent ingestion-specific context among these reports, as documented by others [14]. Vomiting was the most consistently recorded gastrointestinal symptom in kerosene-specific studies. While evidence of vomiting was also identified following jet fuel exposures, the limited number of studies reporting gastrointestinal outcomes in this subset (*N* = 3) precludes meaningful comparisons of gastrointestinal symptoms between fuel types. Similar challenges arise when comparing dermatological outcomes between fuel types, as reports were limited to three kerosene-specific studies and five jet fuel-specific studies; however, contact dermatitis and irritation remained the most common manifestations following both exposures. Changes to skin barrier, while less reported, were also identified with both fuel types. These symptom profiles have been documented in previous reviews, including reports of itching skin, blisters, rashes on hands, chemical allergy, and scaly and weeping skin in workers with greater occupational JP-8 exposure [15]. Moreover, erythema, eczematous lesions and defatting dermatitis were identified in women who regularly handled kerosene [15]. Similar effects, including edema, erythema, dermatitis, and desquamation, have been observed in rats, mice, rabbits and pigs following dermal application of JP-5, JP-8 and Jet A, with symptom severity varying based on duration and concentration of exposure [14].

Little is known of the underlying pathology between kerosene and jet fuel exposures with adverse health outcomes. One experimental study demonstrated jet fuel (Jet A and JP-8) induced production and release of the pro-inflammatory cytokines Tumour Necrosis Factor-alpha (TNF-α) and Chemokine (C-X-C motif) ligand 8 (CXCL8) (also known as Interleukin-8 (IL-8)) by normal human epidermal keratinocytes, which comprise the majority of the epidermis and play a critical role in maintaining the skin’s protective barrier [68]. A key mediator in the development of airway inflammatory diseases [69], TNF-α has been implicated in conditions such as asthma, chronic bronchitis, chronic obstructive pulmonary disease (COPD), acute lung injury and acute respiratory distress syndrome [70]. Similarly, elevated CXCL8 has been observed in respiratory conditions including severe asthma [71] and COPD [72]. TNF-α and/or CXCL8 have also been established in inflammatory processes affecting the neurological, dermatological and gastrointestinal systems [73–75]. The laboratory evidence indicating release of TNF-α and CXCL8 following jet fuel exposure, together with their recognized role in inflammatory processes of bodily systems affected within this review, supports the plausibility that the mechanisms of jet fuel and kerosene toxicity may be mediated in part by these cytokines.

#### Exposure context

Within each body of literature (i.e. kerosene- vs. jet fuel- specific publications), the predominant routes and duration of exposures remained relatively consistent, but differed largely between them. For instance, all kerosene-specific studies involved acute exposure, primarily through ingestion. In contrast, only one jet fuel publication reported an acute event, while the remainder involved chronic occupational exposures (91%). Among these studies, ingestion was rarely observed (18%), while inhalation (82%) and dermal (73%) routes comprised the vast majority of exposures. The rate and extent of TPH absorption varies depending on how the compounds enter the body [24]. Thus, it is possible that while a subset of bodily systems (i.e. respiratory, neurological, gastrointestinal and dermatological) may be more susceptible to these exposures generally, the unique vulnerability and symptom manifestation of each system observed may be influenced in part by the route and duration of exposure.

Health outcomes observed may also reflect differences in chemical composition between fuel types. Among kerosene-specific studies, most involved exposures to pure kerosene, though some (29%) involved kerosene-based products such as lubricants or pesticides. Jet fuel studies predominantly reported on JP-5 or JP-8 specifically (73%), which contain approximately 99.5% kerosene [25]. Considering that jet fuel specific literature is centred primarily on men in occupational settings, the inclusion of kerosene in this review enabled an understanding of potential health outcomes stemming from ingestion related events, in additional settings and population groups like women and children for whom the evidence is particularly scarce in jet fuel research alone. While refinement processes and performance additives create key compositional differences between kerosene and jet fuel, prior research recognizes that their similar chemical and physical properties justify applying toxicological data from one fuel to the other [14, 76].

We cannot attribute the health outcomes observed in this review to pre-combustion forms of kerosene and kerosene-based jet fuel exclusively. Kerosene exposures largely involved acute ingestion in LMIC where kerosene is often used as household fuel. Similarly, jet fuel exposures primarily occurred in occupational settings, with aviation workers experiencing recurrent exposure over several years. In both settings, it is likely that individuals were exposed to post-combustion byproducts, and other co-occurring environmental pollutants; however, the descriptive nature of most included publications limits the consideration of these potential confounders. Despite these limitations, the consistent presence of kerosene as a base compound and the descriptive nature of most publications together highlight key similarities across studies, supporting the potential for shared toxicological patterns such as respiratory, neurological, gastrointestinal and dermatological health implications. Lastly, other system-level health outcomes were rarely documented within either subset. The system level alignment, including those most frequently affected, as well as those absent, suggests that respiratory, neurological, gastrointestinal and dermatological systems may be particularly vulnerable to these exposures, and should be assessed further in future studies.

#### Demographic groups

We intended to evaluate potential differences in health outcomes across sub-populations, including sex and other demographic factors. However, these subgroup analyses were not feasible. Most studies did not provide extractable demographic-specific outcomes. For example, many disclosed the percentage of male versus female participants, but provided outcomes only as aggregate percentages of the total sample without specifying which participants experienced which health outcomes. Thus, it was not possible to determine whether certain symptoms were experienced by some, or all, demographic groups assessed within a given study. The data was often unavailable, or limited, for groups like older adults and pregnant women. Consequently, we were unable to meaningfully conclude whether a given health outcome was disproportionately experienced among women, children or other populations.

### Future studies assessing risk of kerosene and jet fuel on human health

Very few included studies were analytical in design. Of the three that compared outcomes by exposure level (two of good or excellent quality), all focused exclusively on jet fuel [44, 49]. The authors observed increasing JP-8 exposure to be associated with decreased neurocognitive performance (e.g., visual memory and motor speed), poorer central auditory processing, and worse hearing thresholds [44, 49]. Descriptive evidence from jet fuel-specific literature included in this review further supports neurological risks, particularly through reports of auditory impacts, and impaired cognitive function and processing. Several similar effects have also been observed in animals exposed to JP-8, including lethargy, ototoxic hearing loss, central auditory dysfunction, and impaired learning in rats [14, 24]. Comparable neurological outcomes in humans have been documented in prior reviews as well, including nervous system depression, lethargy, coma, drowsiness, convulsions, restlessness and irritability following kerosene ingestion [14, 24]. Together, neurotoxicity appears consistent across analytical and descriptive evidence identified in this review, as well as previous literature on kerosene ingestion, all of which is supported further by findings from animal studies. Efforts should be made to continue exploring the unique risks of jet fuel and kerosene on neurocognitive health. This is of particular importance for exposure during fetal development and early childhood.

Although jet fuel-specific reports typically involved more chronic exposures, their descriptive nature or cross-sectional designs largely prevented the identification of health outcomes requiring longer latency periods. Similarly, kerosene studies often reported on clinical evaluations immediately following poisoning. In the sole cohort study identified in this review, the follow-up period spanned only one work week in order to establish neurological outcomes associated with jet fuel exposures. As a result, the lack of evidence demonstrating cancer, endocrine, developmental, hepatic, immune, reproductive, musculoskeletal and metabolic outcomes is likely indicative of a limited follow up needed to observe these more latent health outcomes, rather than an absence of risk. Future research should prioritize longitudinal designs to better detect longer-latency health effects that may remain undiscovered in short-term studies.

The need to address this research gap is emphasized by the adverse health outcomes undetected by the current review being previously observed in animal studies. Cancerous outcomes such as malignant lymphomas and skin cancer following dermal JP-5 exposure, as well as tumours of the uterus and vagina following kerosene ingestion were reported by toxicological profiles of jet fuel [14, 24]. Reproductive and developmental effects resulting from ingestion and inhalation of JP-8 have also been found in mice, including increased maternal deaths, decreased sperm motility, reduced fetal weight, decreased litter size, and suppressed immune function in pups [14, 15]. Furthermore, decreased body weight - particularly in male, fetal and pregnant rats- appeared in several animal studies following oral and inhalation exposures to both JP-5 and JP-8, but was not identified in this review [14, 24].

Self-reported data informed most included reports, with only three studies conducting formal exposure assessment. Considering that jet fuel and kerosene share complex hydrocarbon mixtures with many other petroleum products, no single compound can indicate their exclusive presence. Although compounds such as naphthalene or benzene can be measured to inform exposure levels, they are widely present in the environment and cannot be used to distinguish contact specifically to pre- versus post- combustion fuel forms [14]. Therefore, future studies should strive to employ a combination of personal sampling, and contextual factors that demonstrate contact with raw fuel specifically. Similarly, the greater part of all health outcomes identified by this review stem from descriptive accounts of clinical evaluations which did not account for confounding variables. Given that most jet fuel-specific literature involved occupational settings, and kerosene-specific literature originated primarily from LMIC, additional environmental exposures, as well as underlying health status, may be unaccounted for. Future studies should address potential confounding or mediating influences of co-occurring pollutants.

The predominant occupational focus among the few analytical studies identified by this review may be due to the recognition of jet fuel as one of the largest chemical exposures facing both military and civilian aviation workers [15]. However, only three studies conducting formal testing of associations between pre-combustion jet fuel on human health have been identified across the review period (2017–2024). Despite the recognized occupational burden of jet fuel across military and non-military settings alike, regulatory exposure thresholds continue to rely heavily on animal models and in vitro toxicological studies. There remains a need for further occupational research to ensure regulatory limits are grounded in accurate and real-world human data. Moreover, recent jet-fuel contamination events, such as the spill at Red Hill on Oʻahu, Hawaiʻi, and in Bucks County, Pennsylvania, highlight the importance of expanding research beyond occupational settings. Future studies should examine the particular risks of non-occupational, and ingestion related exposures as well, as they remain largely unexplored in current research. Efforts should be made to prioritize inclusion of women, children and older adults for whom the evidence is particularly limited.

### Study strengths and limitations

To our knowledge, this is the first systematic review to evaluate the evidence of known and potential health outcomes of exposure to pre-combustion forms of kerosene and kerosene-based jet fuels specifically. This review adhered to PRISMA reporting guidelines as well as OHAT procedures in evidence identification, data extraction and synthesis, and risk of bias appraisals. The participation of four reviewers in article selection, data extraction, quality assessment and risk of bias appraisals decrease the likelihood and influence of bias. While limiting inclusion to English, French, Dutch, Spanish or Portuguese publications may have omitted relevant articles, only one study was excluded during full text screening because of language (German). Additionally, the evaluation of two chemically similar but distinct fuels expanded our evidence base, and enabled observation of a broader range of reported health outcomes, as well as consistencies, or lack thereof, in symptom patterns between fuel types. As jet fuel research primarily involves occupational dermal and inhalation exposures among men of working age, limiting the review to this subset of literature would likely omit a toxicological understanding of additional exposure contexts, particularly related to raw, unburned fuel. With kerosene comprising the vast majority of jet fuel’s composition, its inclusion in this review allows for an evaluation of potential health outcomes stemming from ingestion, non-occupational settings and among women, children and older adults. The wide scope of this review supports a more comprehensive understanding of potential human health outcomes across generally understudied settings and populations in this research area - particularly those exposures occurring in the home and community, and across the lifespan.

Comparing findings among and between groups by age, sex and setting proved difficult given the heterogeneity of fuel type, exposure route and outcomes, as well as the limited number of studies available within each subgroup. While these differences complicate meaningful comparisons across all included studies, consistency in exposure route, setting and age group within each subset of publications (i.e. kerosene- vs. jet fuel- specific studies) allowed for comparisons, a particularly interesting feature of this review. With only 28 included studies - fewer of good or excellent quality - the evidence base is limited, reducing confidence in the patterns observed. Being that the greater part of all included literature consisted of descriptive accounts of health outcomes observed following exposures (e.g., case reports and case series), and formal testing of associations between these variables was rarely conducted, the ability to assess fuel attributable causality from the findings of this review is not possible. The few epidemiological studies conducting assessments of associations between exposure and health effects focused solely on neurological and/or auditory outcomes. Consequently, the evidence of health outcomes within other bodily systems are informed solely by descriptive studies. The inclusion of these descriptive reports however, allowed for a broader examination of potential health outcomes linked to kerosene and jet fuel exposures despite the few analytical studies conducted to date. Given these constraints, a meta-analysis could not be conducted. As a result, reported health outcomes were synthesized at the study level, where larger analytical studies and individual case reports contributed equally to frequency counts. This approach allowed for an assessment of consistency of health outcomes observed following kerosene and/or jet fuel exposures, despite varying study designs and outcome data. Finally, the vast differences in exposure duration between fuel-specific studies provide an insight as to potential health outcomes related to acute versus chronic exposures. These conclusions, however, are challenged by key differences in geographical setting, participant age, exposure routes, dosage levels, as well as the chemical composition of each fuel type. Since reports of kerosene poisoning comprised a larger portion of included reports, the data identified, and subsequent findings, may be moderately weighed in favour of acute symptoms. The urgency to explore health implications of chronic exposure is underscored by the scarcity of longitudinal studies, and lack of health effects typically requiring longer latency periods (e.g., reproductive, developmental, and cancerous outcomes), identified in this systematic review.

A distinct aspect of this review is that it expands the current knowledge-base on health outcomes from these fuels by purposely extracting information on diverse population groups and settings. This contrasts with much of the previous research that has predominantly focused on occupational exposure, limiting knowledge of human health outcomes primarily to men in the military [16, 17]. Recent high-profile drinking water contamination events [1–3, 10, 11] underscore the importance of synthesizing literature on pre-combustion kerosene fuels and evaluating it across diverse populations and settings.

## Conclusion

Considering the vast consumption of kerosene and kerosene-based jet fuels globally, widespread anthropocentric releases of TPHs into the environment, recent water contamination events, and the limitations of current research, further efforts should be made to better understand the health implications of exposure to these raw fuels. The importance of this research is supported by this review, which finds consistent evidence of adverse human health outcomes in various settings and across the lifespan. To this end, evidence generated through this review may inform future epidemiological studies, to better evaluate and discern the cumulative impact of exposure to unburned jet fuel and kerosene on human health outcomes.

## Supplementary Information

Additional File 1.

Additional File 2.

Additional File 3.

Additional File 4.

Additional File 5.

Additional File 6.

Additional File 7.

Additional File 8.

Additional File 9.

Additional File 10.

Additional File 11.

## Data Availability

The datasets supporting the conclusions of this article are included within the article and its additional files.

## Abbreviations

ATSDR: Agency for Toxic Substances and Disease Registry
B/D: Barrels per Day
COPD: Chronic Obstructive Pulmonary Disease
CXCL8: C-X-C motif chemokine Ligand 8
HAP: Household Air Pollution
ICD-10: International Classification of Diseases, Tenth Revision
IL-8: Interleukin 8
JP: Jet Propellant
LMIC: Low-and-middle income countries
OHAT: Office of Health Assessment and Translation
PECO: Population, Exposure, Comparator, Outcome
PH: Petroleum Hydrocarbons
PRISMA: Preferred Reporting Items for Systematic Reviews and Meta-Analyses
PROSPERO: The International Prospective Register of Systematic Reviews
SpO2: Peripheral oxygen saturation
TNF-α: Tumor Necrosis Factor Alpha
TPH: Total Petroleum Hydrocarbon
U.S.: United States of America
