# Supplementary material for: A systematic review of human studies assessing the health effects of unburned kerosene-based jet fuels and products across diverse populations and settings
Source: Environ Health. 2026 Mar 16;25:34. doi: 10.1186/s12940-026-01287-7 (PMC13085620; doi:10.1186/s12940-026-01287-7)
Supplement: Supplementary file 1 — Additional File 1. [file 12940_2026_1287_MOESM1_ESM.docx]

**Additional file 1. PECO Framework Guiding the Systematic Review.** Summary of the Population, Exposure, Comparator, and Outcome (PECO) components used to structure the research question and define the eligibility criteria for this systematic review: “What are the known and/or potential health effects of exposure (oral, dermal and inhalation) to pre-combustion forms of kerosene-based jet fuel, and other kerosene products, in humans across all exposure settings and population groups?”

| PECO Component | Criteria Description |
| --- | --- |
| Population | Humans of all ages and population groups, across all exposure settings. Only in-vivo human studies were included. |
| Exposure | \|  \| \| --- \|   Oral, dermal or inhalation exposure to pre-combustion forms of kerosene-based jet fuel and other kerosene products. |
| Comparator | Unexposed, or lesser exposed, individuals. Studies without comparators were still eligible if they reported on outcomes of exposure. |
| Outcome | Any health outcome. |
