# Supplementary material for: A systematic review of human studies assessing the health effects of unburned kerosene-based jet fuels and products across diverse populations and settings
Source: Environ Health. 2026 Mar 16;25:34. doi: 10.1186/s12940-026-01287-7 (PMC13085620; doi:10.1186/s12940-026-01287-7)
Supplement: Supplementary file 2 — Additional File 2. [file 12940_2026_1287_MOESM2_ESM.docx]

**Additional file 2. Search for Existing Systematic Reviews and Systematic Review Protocols.** This table outlines the search for pre-existing works in fit-per-purpose databases.

| **Database** | **Fields searched** | **Yield - number of reviews** | **Potentially applicable work - number of reviews** | **Applicable works after article review** |
| --- | --- | --- | --- | --- |
| Cochrane | Title/Abstract/Keyword | 0 | 0 | 0 |
| Epistemonikos | Title/Abstract | 32 | 9 | 0 |
| Prospero (with a registration date prior to our review) | Title OR Intervention/Exposure OR Keyword | 20 (3 completed) | 14 | 0 |

**Search terms**

Cochrane Library, Epistemonikos and PROSPERO were searched using the same jet fuel and kerosene exposure terms that were used in the search strategy for the current systematic review. In order not to limit the search, the “AND” health effect terms were left out of the search terms for existing and ongoing systematic reviews. The search terms for each database are listed below.

**Cochrane Library:**

"JP5 jet fuel" OR "S-8 fuel" OR "JP8 aviation fuel" OR "kerosene" OR "jet propellant" OR "jet propellants" OR "aviation fuel" OR "aviation fuels" OR "jet fuel" OR "jet fuels" OR "turbine fuel" OR "turbine fuels" OR "jet propulsion fuel" OR "jet propulsion fuels" OR "Jet A" OR "Jet A-1" OR "Jet B" OR "JP-1" OR" JP1" OR "JP-2" OR "JP2" OR "JP-3" OR "JP3" OR "JP-4" OR "JP4" OR "JP-5" OR "JP5" OR "JP-6" OR "JP6" OR "JP-7" OR "JP7" OR "JP-8" OR "JP8" OR "zip fuel" OR "zip fuels" OR "JPTS" OR "Jet Propellant Thermally Stable" OR "TS-1" in Title Abstract Keyword - in Cochrane Reviews (Word variations have been searched)

**Epistemonikos:**

(title:(("JP5 jet fuel" OR "S-8 fuel" OR "JP8 aviation fuel" OR "kerosene" OR "jet propellant" OR "jet propellants" OR "aviation fuel" OR "aviation fuels" OR "jet fuel" OR "jet fuels" OR "turbine fuel" OR "turbine fuels" OR "jet propulsion fuel" OR "jet propulsion fuels" OR "Jet A" OR "Jet A-1" OR "Jet B" OR "JP-1" OR "JP1" OR "JP-2" OR "JP2" OR "JP-3" OR "JP3" OR "JP-4" OR "JP4" OR "JP-5" OR "JP5" OR "JP-6" OR "JP6" OR "JP-7" OR "JP7" OR "JP-8" OR "JP8" OR "zip fuel" OR "zip fuels" OR "JPTS" OR "Jet Propellant Thermally Stable" OR "TS-1")) OR abstract:(("JP5 jet fuel" OR "S-8 fuel" OR "JP8 aviation fuel" OR "kerosene" OR "jet propellant" OR "jet propellants" OR "aviation fuel" OR "aviation fuels" OR "jet fuel" OR "jet fuels" OR "turbine fuel" OR "turbine fuels" OR "jet propulsion fuel" OR "jet propulsion fuels" OR "Jet A" OR "Jet A-1" OR "Jet B" OR "JP-1" OR "JP1" OR "JP-2" OR "JP2" OR "JP-3" OR "JP3" OR "JP-4" OR "JP4" OR "JP-5" OR "JP5" OR "JP-6" OR "JP6" OR "JP-7" OR "JP7" OR "JP-8" OR "JP8" OR "zip fuel" OR "zip fuels" OR "JPTS" OR "Jet Propellant Thermally Stable" OR "TS-1")))

**PROSPERO - International prospective register of systematic reviews:**((("JP5 jet fuel" OR "S-8 fuel" OR "JP8 aviation fuel" OR "kerosene" OR "jet propellant" OR "jet propellants" OR "aviation fuel" OR "aviation fuels" OR "jet fuel" OR "jet fuels" OR "turbine fuel" OR "turbine fuels" OR "jet propulsion fuel" OR "jet propulsion fuels" OR "Jet A" OR "Jet A-1" OR "Jet B" OR "JP-1" OR "JP1" OR "JP-2" OR "JP2" OR "JP-3" OR "JP3" OR "JP-4" OR "JP4" OR "JP-5" OR "JP5" OR "JP-6" OR "JP6" OR "JP-7" OR "JP7" OR "JP-8" OR "JP8" OR "zip fuel" OR "zip fuels" OR "JPTS" OR "Jet Propellant Thermally Stable" OR "TS-1")):IV)

OR

((("JP5 jet fuel" OR "S-8 fuel" OR "JP8 aviation fuel" OR "kerosene" OR "jet propellant" OR "jet propellants" OR "aviation fuel" OR "aviation fuels" OR "jet fuel" OR "jet fuels" OR "turbine fuel" OR "turbine fuels" OR "jet propulsion fuel" OR "jet propulsion fuels" OR "Jet A" OR "Jet A-1" OR "Jet B" OR "JP-1" OR "JP1" OR "JP-2" OR "JP2" OR "JP-3" OR "JP3" OR "JP-4" OR "JP4" OR "JP-5" OR "JP5" OR "JP-6" OR "JP6" OR "JP-7" OR "JP7" OR "JP-8" OR "JP8" OR "zip fuel" OR "zip fuels" OR "JPTS" OR "Jet Propellant Thermally Stable" OR "TS-1")):KW)

OR

((("JP5 jet fuel" OR "S-8 fuel" OR "JP8 aviation fuel" OR "kerosene" OR "jet propellant" OR "jet propellants" OR "aviation fuel" OR "aviation fuels" OR "jet fuel" OR "jet fuels" OR "turbine fuel" OR "turbine fuels" OR "jet propulsion fuel" OR "jet propulsion fuels" OR "Jet A" OR "Jet A-1" OR "Jet B" OR "JP-1" OR "JP1" OR "JP-2" OR "JP2" OR "JP-3" OR "JP3" OR "JP-4" OR "JP4" OR "JP-5" OR "JP5" OR "JP-6" OR "JP6" OR "JP-7" OR "JP7" OR "JP-8" OR "JP8" OR "zip fuel" OR "zip fuels" OR "JPTS" OR "Jet Propellant Thermally Stable" OR "TS-1")):TI)

**Search Results**

**Cochrane Library:**

This search retrieved 0 records.

**Epistemonikos:**This search retrieved 32 records. Out of these 32 records, nine reviews reported to some extent on jet fuel and/or kerosene products and health effects. These nine reviews can be broadly divided into two categories: those examining indoor/household air pollution (HAP) and those focusing on jet fuel exposure. The majority (6) fall into the former category focusing on indoor/household air pollution (HAP) from cooking and lighting and thus mainly assess exposure to burned fuels. Four of these reviews focusing on HAP also combine kerosene exposure with exposure to other polluting fuels such as coal and biomass. Five of the six HAP studies focus on a limited number of health outcomes. The three studies falling into the second category reporting on jet fuel exposure focus on occupational and/or military populations and two of the three studies limit to a specific health outcome.

| **Reference** | **Focus on all health effects of pre-combustion jet fuel and/or kerosene products in the general population?** |
| --- | --- |
| D Vincent-Hall T, G Bergeron J, E Eftim S, J Lindahl A, R Weinberger K, E Haver C, and J Snow S. 2025. “Health Effects of Occupational Exposure to Jet Fuels Used in the Military: A Systematic Review of the Epidemiologic Literature.” Environment international 196: 109278. doi:10.1016/j.envint.2025.109278. | No |
| Farooq, Z, E Wetterlund, S Mesfun, and E Furusjö. 2025. “Uncovering the Economic Potential of Sustainable Aviation Fuel Production Pathways: A Meta-Analysis of Techno-Economic Studies.” *ENERGY CONVERSION AND MANAGEMENT* 341. doi:10.1016/j.enconman.2025.120076. | No |
| Ismail, R., M. Alherbawi, S. Mariyam, G. Gordon, and T. Al-Ansari. 2025. “Catalytic Pathways towards Sustainable Aviation Fuel Production from Waste Biomass: A Systematic Review.” *Chemical Engineering Journal Advances* 24. doi:10.1016/j.ceja.2025.100927. | No |
| Xu, Y., Y. Zhang, X. Deng, S.-Y. Lee, K. Wang, and L. Li. 2025. “Bibliometric Analysis and Literature Review on Sustainable Aviation Fuel (SAF): Economic and Management Perspective.” *Transport Policy* 162: 296–312. doi:10.1016/j.tranpol.2024.11.014. | No |
| Kurniawan, T.A. 2025. “Uncovering the Potential of Biomass from Agricultural Waste as Sustainable Biofuel in Aviation Industry to Promote Net Zero Emissions: A Critical Review.” *BioResources* 20(2). doi:10.15376/biores.20.2.Kurniawan. | No |
| Gyandoh, E., and J. Gomez. 2025. “Techno-Economic Analysis (TEA) of Civilian Sustainable Aviation Fuel (SAF) – A Systematic Review of Hydrotreated Esters and Fatty Acids (HEFA) and Lignocellulosic Biomass Conversion (LCBC) Strategies.” *Applied Energy* 399. doi:10.1016/j.apenergy.2025.126421. | No |
| Raihan, A. 2025. “Sustainable Aviation: A Critical Review of Policies, Technologies, and Future Pathways.” *Journal of the Air Transport Research Society* 5. doi:10.1016/j.jatrs.2025.100080. | No |
| Bostanci N, Manoil D, Van Holm W, Belibasakis GN, and Teughels W. 2025. “Microbial Markers for Diagnosis and Risk Assessment for Periodontal Diseases: A Systematic Literature Search and Narrative Synthesis.” *Journal of clinical periodontology* 52(S29): 125–54. doi:10.1111/jcpe.14183. | No |
| Derebe, B., A. Alemu, and Z. Asfaw. 2025. “Fuelwood Dependence and Alternative Energy Sources in Ethiopia: A Systematic Review.” *Discover Sustainability* 6(1). doi:10.1007/s43621-024-00721-5. | No |
| Yavuz C, Ravat Z, León MDA, Lee S, Fernandes P, Reifmesser Q, Gaved FE, et al. 2025. “Improving Energy Access, Climate and Socio‐Economic Outcomes Through Off‐Grid Electrification Technologies: A Systematic Review.” *Campbell Systematic Reviews* 21(3): 1–52. doi:10.1002/cl2.70060. | No |
| Yaşar Dinçer FC, Yirmibeşoğlu G, Bilişli Y, Arık E, and Akgün H. 2024. “Trends and Emerging Research Directions of Sustainable Aviation: A Bibliometric Analysis.” *Heliyon* 10(11): e32306. doi:10.1016/j.heliyon.2024.e32306. | No |
| Daba C, Asmare L, Demeke Bayou F, Arefaynie M, Mohammed A, Tareke AA, Keleb A, et al. 2024. “Exposure to Indoor Air Pollution and Adverse Pregnancy Outcomes in Low and Middle-Income Countries: A Systematic Review and Meta-Analysis.” *Frontiers in public health* 12: 1356830. doi:10.3389/fpubh.2024.1356830. | No |
| Yoon SH, Huh BK, Abdi S, and Javed S. 2024. “The Efficacy of High-Intensity Laser Therapy in Wound Healing: A Narrative Review.” *Lasers in medical science* 39(1): 208. doi:10.1007/s10103-024-04146-4. | No |
| Puzzolo E, Fleeman N, Lorenzetti F, Rubinstein F, Li Y, Xing R, Shen G, et al. 2024. “Estimated Health Effects from Domestic Use of Gaseous Fuels for Cooking and Heating in High-Income, Middle-Income, and Low-Income Countries: A Systematic Review and Meta-Analyses.” *The Lancet. Respiratory medicine*. doi:10.1016/S2213-2600(23)00427-7. | No |
| Mehta J, Eaton C, AlAmri M, Lin GH, and Nibali L. 2023. “The Association between Aggregatibacter Actinomycetemcomitans JP2 Clone and Periodontitis: A Systematic Review and Meta-Analysis.” *Journal of periodontal research*. doi:10.1111/jre.13102. | No |
| El Hayek S, Cherro M, El Harake N, and Ghossoub E. 2023. “Self-Immolation in the Arab World: A Systematic Review.” *Burns : journal of the International Society for Burn Injuries* 49(4): 757–69. doi:10.1016/j.burns.2022.10.001. | No |
| Ahmed DR. 2023. “The Epidemiology of Self-Immolation in Iraq: A Systematic Review.” *The International journal of social psychiatry*: 207640231168787. doi:10.1177/00207640231168787. | No |
| Luo M, Liu T, Ma C, Fang J, Zhao Z, Wen Y, Xia Y, Zhao Y, and Ji C. 2023. “Household Polluting Cooking Fuels and Adverse Birth Outcomes: An Updated Systematic Review and Meta-Analysis.” *Frontiers in public health* 11: 978556. doi:10.3389/fpubh.2023.978556. | No |
| Khzam N, Miranda LA, Kujan O, Shearston K, and Haubek D. 2022. “Prevalence of the JP2 Genotype of Aggregatibacter Actinomycetemcomitans in the World Population: A Systematic Review.” *Clinical oral investigations*. doi:10.1007/s00784-021-04343-3. | No |
| Younger A, Alkon A, Harknett K, Jean Louis R, and Thompson LM. 2022. “Adverse Birth Outcomes Associated with Household Air Pollution from Unclean Cooking Fuels in Low- and Middle-Income Countries: A Systematic Review.” *Environmental research* 204(Pt C): 112274. doi:10.1016/j.envres.2021.112274. | No |
| Tayfur A, Haque A, Salles JI, Malliaras P, Screen H, and Morrissey D. 2021. “Are Landing Patterns in Jumping Athletes Associated with Patellar Tendinopathy? A Systematic Review with Evidence Gap Map and Meta-Analysis.” *Sports medicine (Auckland, N.Z.)*. doi:10.1007/s40279-021-01550-6. | No |
| Lee KK, Bing R, Kiang J, Bashir S, Spath N, Stelzle D, Mortimer K, et al. 2020. “Adverse Health Effects Associated with Household Air Pollution: A Systematic Review, Meta-Analysis, and Burden Estimation Study.” *The Lancet. Global health* 8(11): e1427–34. doi:10.1016/S2214-109X(20)30343-0. | No |
| Ishikawa, T, H Uetake, K Murotani, T Kobunai, M Ishiguro, S Matsui, and K Sugihara. 2016. “Genome-Wide DNA Copy-Number Analysis in ACTS-CC Trial of Adjuvant Chemotherapy for Stage III Colonic Cancer.” *ANTICANCER RESEARCH* 36(3): 853–60. | No |
| Warner R, Fuente A, and Hickson L. 2015. “Jet Fuel, Noise, and the Central Auditory Nervous System: A Literature Review.” *Military medicine* 180(9): 950–55. doi:10.7205/MILMED-D-14-00733. | No |
| Bruce N., Pope D., Rehfuess E., Balakrishnan K., Adair-Rohani H., and Dora C. 2015. “WHO Indoor Air Quality Guidelines on Household Fuel Combustion: Strategy Implications of New Evidence on Interventions and Exposure-Risk Functions.” *Atmospheric Environment* 106((Bruce N., ngb@liv.ac.uk; Pope D.) Department of Public Health and Policy, University of Liverpool, United Kingdom): 451–57. doi:10.1016/j.atmosenv.2014.08.064. | No |
| Kurmi OP, Sadhra CS, Ayres JG, and Sadhra SS. 2014. “Tuberculosis Risk from Exposure to Solid Fuel Smoke: A Systematic Review and Meta-Analysis.” *Journal of epidemiology and community health* 68(12): 1112–18. doi:10.1136/jech-2014-204120. | No |
| Kurmi, O., C. Sadhra, J. Ayres, and S. Sadhra. 2013. “Tuberculosis Risk from Exposure to Solid Fuel Smoke: A Systematic Review and Meta-Analysis.” *European Respiratory Journal* 42. http://www.epistemonikos.org/documents/fba5fa81fd859895fabf24e97d9e702ca4c521a1. | No |
| Golshan A, Patel C, and Hyder AA. 2013. “A Systematic Review of the Epidemiology of Unintentional Burn Injuries in South Asia.” *Journal of public health (Oxford, England)* 35(3): 384–96. doi:10.1093/pubmed/fds102. | No |
| Ansell J, Mason J, Warren N, Donnelly P, Hawkes N, Dolwani S, and Torkington J. 2012. “Systematic Review of Validity Testing in Colonoscopy Simulation.” *Surgical endoscopy* 26(11): 3040–52. doi:10.1007/s00464-012-2332-2. | No |
| Jahn CA. 2010. “The Dental Water Jet: A Historical Review of the Literature.” *Journal of dental hygiene : JDH / American Dental Hygienists’ Association* 84(3): 114–20. | No |
| Hernández Contreras, Natividad, Yalina Chang Camero, Yarina Santana Suárez, Elizabeth Machado Martínez, Alicia M Martínez Izquierdo, and Lourdes de la C Pui Vázquez. 2010. “Deliberate Use of Several Products for Pediculus Capitis (De Geer, 1778 ) Control by Parents or Tutors of Elementary School Children.” *Rev. cuba. med. trop* 62(2): 119–24. | No |
| Ryder SJ, Crawford PI, and Pethybridge RJ. 1997. “Is Testicular Cancer an Occupational Disease? A Case-Control Study of Royal Naval Personnel.” *Journal of the Royal Naval Medical Service* 83(3): 130–46. | No |

**PROSPERO:**This search retrieved 20 records of which three were listed as completed and 17 were listed as ongoing. Out of these 20 records, 14 plan to assess, to some extent, kerosene and health effects. The large majority (13) of the registered reviews plan to look at HAP and thus will mainly assess exposure to burned fuels. In addition, kerosene is part of a number of polluting fuels used for indoor cooking or lighting that will be assessed in these reviews. The remaining record plans to conduct a systematic review on kerosene poisoning in children. This record is not marked as completed and a published article could not be found. The results may be informative to answering part of the research question posed in the current systematic review but it will not encompass the full scope of the research question as it is limited to kerosene and children only and may not cover all exposure routes.

| **PROSPERO Record Reference** | **Focus on all health effects of pre-combustion jet fuel and/or kerosene products in the general population** |
| --- | --- |
| Siqi Cheng, Yan Dou, Yuxin Jin, Yan Zhao, Yaxin Duan, Hanlei Pei, Peiyuan Lv. Long-term PM2.5 exposure and dementia: A systematic review and meta-analysis. PROSPERO 2024 Available from <https://www.crd.york.ac.uk/PROSPERO/view/CRD42020166072> | No |
| Utsav Parekh, Abhishek Das, Siddhartha Dutta, Sanjay Gupta. Kerosene Poisoning in Children: A Systematic Review and Quantitative Analysis of Literature.. PROSPERO 2024 Available from <https://www.crd.york.ac.uk/PROSPERO/view/CRD42024511122> | No |
| Kingsley Boakye, Bernard Asamoah Barnie, Arti Singh, Daniel Boateng, Ellis Owusu-Dabo, George Downward, Kerstin Klipstein-Grobusch. Household fuel consumption and cardiometabolic health in sub-Saharan Africa: A Systematic Review. PROSPERO 2024 Available from <https://www.crd.york.ac.uk/PROSPERO/view/CRD42024538239> | No |
| Lily Yan, Miranda Metz, Anju Ogyu, Michelle Demetres, Jennifer Peel, Jack Caravanos, Margaret McNairy. Lead reduction interventions in low- and middle-income countries: systematic review protocol. PROSPERO 2024 Available from <https://www.crd.york.ac.uk/PROSPERO/view/CRD42024547822> | No |
| Suvarna Jyothi Kantipudi, Jayakumar Menon. Household Air Pollution and Dementia Risk in Low- and Middle-Income Countries -A Systematic Review of literature. PROSPERO 2024 Available from <https://www.crd.york.ac.uk/PROSPERO/view/CRD42024560164> | No |
| Aziza Seitova, Aiperi Asanbek kyzy, Nobutoshi Nawa, Roman Kalmatov, Takeo Fujiwara. Air pollution and adverse health outcomes in Central Asia: a systematic review and meta-analysis. PROSPERO 2024 Available from <https://www.crd.york.ac.uk/PROSPERO/view/CRD42024570959> | No |
| Elizabeth McClure, Chanese Forte', Carrie Badlwin-SoRelle, Lilly Adams, Joy McNally, Olivia Vaz, Ayla Le. Documented health impacts of living near the Hanford nuclear facility. PROSPERO 2024 Available from<https://www.crd.york.ac.uk/PROSPERO/view/CRD42024613922> | No |
| Bavapriya Iyngararasan, Salma Ayis, Seeromanie Harding. Adverse health effects associated with household air pollution: An updated systematic review, meta-analysis, and burden estimation with consideration of structural determinants of health. PROSPERO 2024 Available from <https://www.crd.york.ac.uk/PROSPERO/view/CRD42024628318> | No |
| EYASU LAKE, Guy Marks, Christine Cowie. The effect of air pollution on morbidity and mortality among children aged under 5 years in sub-Saharan Africa: Systematic Review. PROSPERO 2024 Available from <https://www.crd.york.ac.uk/PROSPERO/view/CRD42023470010> | No |
| Ashley Younger, Abbey Alkon, Lisa Thompson, Kristen Harknett, Roseline Jean Louis. Adverse Birth Outcomes Associated with Household Air Pollution from Unclean Cooking Fuel in Low- and Middle-Income Countries: A Systematic Review. PROSPERO 2024 Available from <https://www.crd.york.ac.uk/PROSPERO/view/CRD42020152333> | No |
| Chao Ji, mengrui Luo. Household Polluting Cooking Fuels and Adverse Birth Outcomes: A Systematic Review and Meta-Analysis. PROSPERO 2024 Available from <https://www.crd.york.ac.uk/PROSPERO/view/CRD42021269660> | No |
| Daniel Bogale, Luke Knibbs. Household air pollution and health outcomes: a systematic review of global epidemiological evidence from the Demographic and Health Surveys (DHS). PROSPERO 2024 Available from <https://www.crd.york.ac.uk/PROSPERO/view/CRD42019137937> | No |
| Dan Pope, Nigel Fleeman, Emily Nix, Elisa Puzzolo, Gouefeng Shen, Jessica Lewis, Lydia Abebe, Heather Adair-Rohani. Health effects of liquid and gaseous fuel use for household cooking, heating, and lighting systematic review and meta-analysis. PROSPERO 2024 Available from<https://www.crd.york.ac.uk/PROSPERO/view/CRD42021227092> | No |
| Sathya Aithal, Shireen Gill, Om Kurmi. The effects of household air pollution (HAP) from solid fuel burning on the risk of asthma, and acute lower respiratory tract infections, and lung function in children: a systematic review and meta-analysis. PROSPERO 2024 Available from <https://www.crd.york.ac.uk/PROSPERO/view/CRD42021236671> | No |
| Luigi Nibali, Jaimini Mehta, Carolyn Eaton. Prevalence and predictive ability for periodontal progression of the JP2 clone of Aggregatibacter actinomycetemcomitans: a systematic review. PROSPERO 2024 Available from <https://www.crd.york.ac.uk/PROSPERO/view/CRD42021244128> | No |
| Akorede Adekoya, Kurmi Om, Adenike Adeyemi. Effects of household air pollution (HAP) on cardiovascular diseases in low and middle income countries: a systematic review and meta-analysis.. PROSPERO 2024 Available from <https://www.crd.york.ac.uk/PROSPERO/view/CRD42021248800> | No |
| Nabil Khzam, Leticia Algarves Miranda, Omar Kujan, Kate Shearston, Dorte Haubek. The prevalence of the JP2 genotype of Aggregatibacter actinomycetemcomitans in the world population: Systematic review. PROSPERO 2024 Available from <https://www.crd.york.ac.uk/PROSPERO/view/CRD42021264375> | No |
| Christine LOVERA. Dihydropyrimidine dehydrogenase (DPD) deficiency screening to avoid severe fluoropyrimidine-related toxicities: meta-analyses and assessment of current practices in France and search for a multi-gene signature to secure fluorpyrimidine-based chemotherapies in colorectal cancer patient. PROSPERO 2024 Available from <https://www.crd.york.ac.uk/PROSPERO/view/CRD42017058148> | No |
| Florence Omuronji. An updated systematic review on Household Air Pollution as a Risk Factor for Eye Disease.. PROSPERO 2024 Available from <https://www.crd.york.ac.uk/PROSPERO/view/CRD42020193675> | No |
| Christina Ma, Christopher Chiang, Kristen Viaje, Om P Kurmi. Relationship between exposure to household air pollution and asthma, acute lower respiratory infections and lung function in children: a systematic review and meta-analysis. PROSPERO 2024 Available from <https://www.crd.york.ac.uk/PROSPERO/view/CRD42018094283> | No |
