# Supplementary material for: A systematic review of human studies assessing the health effects of unburned kerosene-based jet fuels and products across diverse populations and settings
Source: Environ Health. 2026 Mar 16;25:34. doi: 10.1186/s12940-026-01287-7 (PMC13085620; doi:10.1186/s12940-026-01287-7)
Supplement: Supplementary file 3 — Additional File 3. [file 12940_2026_1287_MOESM3_ESM.docx]

**Additional file 3. Terms Screened and Excluded from Search Strategy.** This table lists terms not included in search strategy after failing to retrieve additional results during database searches.

| Database | Search terms |
| --- | --- |
| PubMed | "acute effects”, angina, anxiety, asthma, blisters, “blurr* vision”, body weight, bronchitis, bronchoconstriction, cancer, cardio*, cancerous, cardiovascular, chest tightness, “chronic obstructive pulmonary disease”, “chronic illness”, coenzymes, congenital abnormalities, congenital diseases, connective tissue, confusion, convulsions, dermatitis, developmental, developmental disorder, diarrhea, digestive, dyspnea, endocrine, emphysema, estrogen, “eye disease”, faint*, “heart disease”, "hemic diseases", hemorrhage, "hereditary diseases", "hereditary abnormalities", histopatholog*, hormonal, “immunotoxicity”, imbalance, inflammation, irrita*, liver injury, lightheaded*, lymph, lymphatic diseases, mental disorder, “mental health”, mutation, musculoskeletal, nausea, neoplasm, "neonatal diseases", "neonatal abnormalities", “nervous system”, neurological, “ocular”, ototoxic, pulmonary lesions, pregnancy complications, pulmonary, quality of life, remember*, reproductive, respiratory, seizures, skin, stomatognathic, systemic effects, tachycardia, tremor, “toxicology”, “toxic effects”, “toxicological effects”, urogenital, "urogenital diseases", “vomit*”, “walk*”, wheez* |
| Web of Science | “acute effects”, angina, asthma, anxiety, bronchitis, blisters, “blurr* vision”, "body weight", bronchoconstriction, cardiovascular, "connective tissue", cancerous, cardio*, "chest tight*", cough*, "congenital abnormalities", "congenital diseases", coenzymes, cerebrovascular, “chest tightness”, “chronic obstructive pulmonary disease”, convulsions, “chronic disease”, “chronic effects”, "chronic illness", "developmental disorder", dental, developmental, derma*, dyspnea, dizz*, etiolog*, endocrin*, eyes, "eye disease", “eye diseases”, emphysema, faint*, forget*, gastro*, gastrointestinal, gestation, genomics, hear*, hepatic, hemorrhage, histopatholog*, "hemic diseases", "hereditary diseases", "hereditary abnormalities", “health outcome”, itch*, "immune system", kidney*, "lymphatic diseases", "liver injury", neoplasm, "neonatal diseases", "neonatal abnormalities", musculoskeletal, “mental illness”, morbidit*, “mental health”, “mental disorder”, mutagen, mutation, neurolog*, "nervous system", ocular, "occupational accidents", “out of breath”, patholog*, pulmonary, "pregnancy complications", remember*, reproductive, rash, sex, “stomatognathic diseases", symptom,"toxic* effects", urogenital diseases, vision, vertigo, wheez*, worry |
