# Supplementary material for: A systematic review of human studies assessing the health effects of unburned kerosene-based jet fuels and products across diverse populations and settings
Source: Environ Health. 2026 Mar 16;25:34. doi: 10.1186/s12940-026-01287-7 (PMC13085620; doi:10.1186/s12940-026-01287-7)
Supplement: Supplementary file 5 — Additional File 5. [file 12940_2026_1287_MOESM5_ESM.docx]

**Additional file 5. Quality Assessment Framework for Cross-Sectional Studies.** Distinct quality assessment criteria for cross-sectional studies.

Max Score: 21

| **Item** | **Criteria** | **Answers** |
| --- | --- | --- |
| **Reporting** | | |
| 1 | *Is the hypothesis/aim/objective of the study clearly described?* | Yes = 1  No = 0 |
| 2 | *Are the main outcomes to be measured clearly described in the Introduction or Methods section?* If the main outcomes are first mentioned in the Results section, the question should be answered no. | Yes = 1  No = 0 |
| 3 | *Are the characteristics of the participants included in the study clearly described?*  Characteristics of participants are included. | Yes = 1  No = 0 |
| 4 | *Are the distributions of principal confounders in each group of subjects to be compared clearly described?*  A list of principal confounders is provided.^a^ | Yes = 2  Partially = 1  No = 0 |
| 5 | *Are the main findings of the study clearly described?*  Simple outcome data (including denominators and numerators) should be reported for all major findings so that the reader can check the major analyses and conclusions. (This question does not cover statistical tests which are considered below). | Yes = 1  No = 0 |
| 6 | *Does the study provide estimates of the random variability in the data for the main outcomes?*  In non-normally distributed data the interquartile range of results should be reported. In normally distributed data the standard error, standard deviation or confidence intervals should be reported. If the distribution of the data is not described, it must be assumed that the estimates used were appropriate and the question should be answered yes^b^ | Yes = 1  No = 0 |
| 7 | *Have actual probability values been reported (e.g. 0.035 rather than <0.05) for the main outcomes except where the probability value is less than 0.001?^c^* | Yes = 1  No = 0 |
| **External validity** | | |
| 8 | *Were the subjects asked to participate in the study representative of the entire population from which they were recruited?*  The study must identify the source population for participants and describe how they were selected. Participants would be representative if they comprised the entire source population, an unselected sample of consecutive participants, or a random sample. Random sampling is only feasible where a list of all members of the relevant population exists. Where a study does not report the proportion of the source population from which the participants are derived, the question should be answered as unable to determine. | Yes = 1  No = 0  Unable to determine = 0 |
| 9 | *Were those subjects who were prepared to participate representative of the entire population from which they were recruited?*  The proportion of those asked who agreed should be stated. Validation that the sample was representative would include demonstrating that the distribution of the main confounding factors was the same in the study sample and the source population. | Yes = 1  No = 0  Unable to determine = 0 |
| **Internal validity - bias** | | |
| 10 | *If any of the results of the study were based on “data dredging”, was this made clear?*  Any analyses that had not been planned at the outset of the study should be clearly indicated. If no retrospective unplanned subgroup analyses were reported, then answer yes. | Yes = 1  No = 0  Unable to determine = 0 |
| 11 | *Were the statistical tests used to assess the main outcomes appropriate?*  The statistical techniques used must be appropriate to the data. For example nonparametric methods should be used for small sample sizes. Where little statistical analysis has been undertaken but where there is no evidence of bias, the question should be answered yes. If the distribution of the data (normal or not) is not described it must be assumed that the estimates used were appropriate and the question should be answered yes. | Yes = 1  No = 0  Unable to determine = 0 |
| 12 | *Were the main outcome measures used accurate (valid and reliable)?*  For studies where the outcome measures are clearly described, the question should be answered yes. For studies which refer to other work or that demonstrates the outcome measures are accurate, the question should be answered as yes. | Yes = 1  No = 0  Unable to determine = 0 |
| **Internal validity – confounding (selection bias)** | | |
| 13 | *Were the participants recruited from the same population?*  This question should be answered yes when the sample was drawn from the same source population. In multi-site studies, answer yes when participants are recruited from similar source populations across all sites. If participant characteristics are not provided, this question should be answered, unable to determine. | Yes = 1  No = 0  Unable to determine = 0 |
| 14 | *Were study subjects recruited over the same period of time?* For a study which does not specify the time period over which participants were recruited, the question should be answered as unable to determine.^d^ | Yes = 1  No = 0  Unable to determine = 0 |
| 15 | *Was there adequate adjustment for confounding in the analyses from which the main findings were drawn?*  In non-randomized studies if the effect of the main confounders was not investigated or confounding was demonstrated but no adjustment was made in the final analyses the question should be answered as no.^d^ | Yes = 1  No = 0  Unable to determine = 0 |
| **Power** |  |  |
| 16* | *Was a power analysis performed?*^d^ | Yes = 1  No = 0  Unable to determine = 0 |
| **Exposure Assessment** | | |
| 17 | *Was the exposure of interest the focus of the study?*  If the exposure was the main variable tested, this question should be answered yes. If the exposure of interest was one of many variables tested, answer no. | Yes = 1  No = 0 |
| 18 | *Were the exposure assessment methods used objective and precise?*  If the study utilized direct methods to measure exposure levels such as biomonitoring or personal samplers, this question should be answered yes. If the study utilized indirect methods such as questionnaires or self-reported data to infer exposure levels, this question should be answered partially. If the study relied on proxies such as job titles or time spent near exposure source to attribute exposure levels, answer no. | Yes = 2  Partially = 1  No = 0 |
| 19 | *Is exposure assessed in a way that allows for dose-response conclusions?*  If the study assessed outcomes of exposure at several levels of varying intensities or concentrations (ex. low, medium and high exposure), answer yes.  If the study categorized exposure into broad groups like “high” and “low” without precise measurements or without including dose-response information, the question should be answered no. | Yes = 1  No = 0 |

*Modified from the original form published by Downs and Black & Carvajal et al., 2025
