# Supplementary material for: A systematic review of human studies assessing the health effects of unburned kerosene-based jet fuels and products across diverse populations and settings
Source: Environ Health. 2026 Mar 16;25:34. doi: 10.1186/s12940-026-01287-7 (PMC13085620; doi:10.1186/s12940-026-01287-7)
Supplement: Supplementary file 6 — Additional File 6. [file 12940_2026_1287_MOESM6_ESM.docx]

**Additional file 6.** **Quality Assessment Framework for Case Reports & Case Series.** Distinct quality assessment criteria for case reports & case series.

Max Score = 10

| **Item** | **Criteria** | **Answers** |
| --- | --- | --- |
| **Reporting** | | |
| 1 | *Were the patient's demographic characteristics clearly described?*  Does the case report clearly describe the patient's age, sex, race, medical history, diagnosis and prognosis? The setting and context may also be described. | Yes = 2 Some described = 1  No = 0 |
| 2 | *Was the presenting clinical condition of the patient on presentation clearly described?* The presenting clinical condition of the patient should be described in detail including the uniqueness of the condition/disease, symptoms, frequency and severity. The case report should also be able to present whether differential diagnoses was considered. | Yes = 2 Some described = 1  No = 0 |
| 3 | *Were diagnostic tests or assessment methods and the results clearly described?*  A reader of the case report should be provided sufficient information to understand how the patient was assessed. The case report should provide a clear description of various diagnostic tests used. | Yes = 1  No = 0 |
| 4 | *Was the intervention(s) or treatment procedure(s) clearly described?*  The report should describe the treatment/intervention protocol in detail; for e.g. in pharmacological management of dental anxiety - the type of drug, route of administration, drug dosage and frequency, and any side effects. | Yes = 2  Some described = 1  No = 0 |
| 5 | *Was the post-intervention clinical condition clearly described?*  The case report should clearly describe the clinical condition post-intervention in terms of the presence or lack thereof symptoms. | Yes = 1  No = 0 |
| 6 | *Were adverse events (harms) or unanticipated events identified and described?*  Any adverse events after administering treatment/intervention/drug are clearly documented and described. If authors mention no adverse events occurred after treatment/intervention, then answer yes. | Yes = 1  No = 0 |
| 7 | *Does the case report provide takeaway lessons?*  Case reports should summarize key lessons learned from a case in terms of the background of the condition/disease and clinical practice guidance for clinicians when presented with similar cases. | Yes = 1  No = 0 |

Modified from: Moola S, Munn Z, Tufanaru C, Aromataris E, Sears K, Sfetcu R, Currie M, Qureshi R, Mattis P, Lisy K, Mu P-F. Chapter 7: Systematic reviews of etiology and risk. In: Aromataris E, Munn Z (Editors). Joanna Briggs Institute Reviewer's Manual. The Joanna Briggs Institute, 2017. Available from https://reviewersmanual.joannabriggs.org/
