# Supplementary material for: A systematic review of human studies assessing the health effects of unburned kerosene-based jet fuels and products across diverse populations and settings
Source: Environ Health. 2026 Mar 16;25:34. doi: 10.1186/s12940-026-01287-7 (PMC13085620; doi:10.1186/s12940-026-01287-7)
Supplement: Supplementary file 7 — Additional File 7. [file 12940_2026_1287_MOESM7_ESM.docx]

**Additional file 7. Categorization of Extracted Health Effects by Bodily System**. Unique health effects listed as stated in original studies, categorized by clinical similarity within bodily systems.

| **Bodily System** | **Symptom Category** | **Raw Symptoms Grouped** |
| --- | --- | --- |
| **Respiratory** | Abnormal Breath Sounds | Bibasal coarse crackles; bilateral coarse crackles; breath sounds decrease with inspiratory rales and crackle on right side; crepitations; decreased breath sounds; decreased breath sounds over right hemithorax; diminished breath sounds; fine crepitations heard in middle and lower lungs; inspiratory rales and crackles bilaterally; noisy breathing; transmitted sounds |
|  | Chest Discomfort | Angina*; burning lungs; chest pressure; chest tightness/pain/angina; pleuritic pain; sharp right sided and pleuritic chest pain |
|  | Cough | Cough; dry cough |
|  | Infectious/Inflammatory Lung Conditions | Aspiration pneumonia; chemical pneumonitis; pneumonitis; pneumonia; lipid pneumonia |
|  | Laboured Breathing | Breathlessness; bronchial breathing; dyspnea; laboured breathing; shortness of breath; tachypnea; tachypneic; tachypnoea; rapid breathing; wheeze |
|  | Tissue/Mechanical Impacts | Bilateral dependent dense consolidation and multiple pneumatoceles (some showing air fluid levels); bilateral infiltrates; bronchiovascular markings; bronchopleural Fistula (BPF); burn injuries in respiratory tract; consolidation in bilateral lower lobes; costophrenic angle blunting with thickening of right minor fissure; decreased air entry to right lung; consolidation in right middle and left lower lobes; edematous and erythematous changes in right middle and lower lobe with grayish secretions; emphysematous changes; empyema; right middle lobe/right lower lobe consolidation; ill-defined areas of ground-glass opacities with superimposed interlobular septal thickening (crazy-paving pattern) with predominantly lower lobar distribution and low attenuating (lipid-containing) opacities; increased bilateral hilar shadow; infiltration in both lower lobes with low-density area in infiltrative shadow; loculated collection in right middle and lower lobe with pleural thickening; mild right sided pleural effusion; necrotic consolidation; ground glass opacity (GGO) and bronchial wall thickening in right middle/lower lobe; patchy air space consolidation in right lower zone of lung; patchy infiltrates in bilateral lower zones; pleural collection along right posterior chest wall with air fluid level and posterior basal subsegmental bronchus leading to collection; surrounding subsegmental regional consolidation with mild contralateral mediastinal shift; pleural effusion on right side with collapse; pneumothorax; pus pockets found in middle and lower lobe of right lung right infiltrates; right pleural effusion; thick pus; volume loss with air-fluid level in retrocardiac region on right side |
|  | Upper Respiratory Irritation | Burning nose or throat; nose bleeds; runny nose; sinus congestion; sinus infections; sneezing; upper respiratory infection |
|  | Other Respiratory Impacts | Acute respiratory distress syndrome; decline in exercise tolerance; respiratory distress |
| **Neurological** | Auditory Impacts | Changes to audiometric thresholds; difficulty in listening situations; ear pain; ear pressure; lower speech intelligibility in noise; muffled hearing; ringing in ears; worse hearing thresholds; non-infectious otitis externa |
|  | Cognitive Function & Processing | Confusion; confusion; difficulty concentrating; difficulty remembering; mild right frontal hypoperfusion; performance with non-dominant hand on Grooved pegboard task; poor central auditory processing (performances in visual memory and motor speed); visual memory |
|  | Equilibrium & Spatial Disorientation | Bilateral vestibular dysfunction on rotational chair test; dizziness; dizzy; fall on Romberg test; feeling of drifting to one side of hallway, overcorrecting and stumbling; general impairment of equilibrium and predominant vestibular deficit pattern; giddiness; imbalance; impaired equilibrium; persistent dizziness; reduced caloric vestibular responses; reduced gain with sinusoidal rotation; sensation of spinning; sensation of tilting; worsening dizziness |
|  | Head Pain & Discomfort | Headache; head fullness |
|  | Impaired Consciousness | Altered sensorium (irritability, lethargy, drowsiness); delirium; drowsiness; fainting; fatigue; loss of consciousness; stupor; unusual sleepiness |
|  | Mood & Affective Symptoms | Agitation/irritability; anxiety; depression; difficulty sleeping; feeling depressed; paranoia; restlessness; tension/nervousness |
|  | Seizures & Convulsions | Convulsions; seizures |
|  | Sensory Impairment | Feeling numb; loss of pain, cold, hunger sensations; numbness |
| **Gastrointestinal** | Abdominal Pain | Abdominal pain; epigastric pain |
|  | Appetite Decline | Appetite decline; decline in appetite/oral intake; loss of appetite |
|  | Diarrhea | Diarrhea |
|  | Nausea | Nausea |
|  | Vomiting | Vomiting |
|  | Other | Pancreatitis, double gastric fluid level |
| **Dermatological** | Contact Dermatitis/Irritation | Cracking; crusting; dry or itchy skin; edema; erythema; hyperkeratosis; itching; perianal inflammation; rash; scaling; skin blisters; skin irritation or burning; skin rash; swelling; weeping; worsening pruritic rash |
|  | Changes to Skin Barrier | Burn injuries; desquamation and wet-appearing skin with debris consisting of large amounts of gray to whitish desquamating skin; rectal burns; transient whitening of skin; whitish appearing excoriations |
|  | Features of Infection/Abscess | New onset anal ulcers; abscess-like presentation; phlegmonous changes; purulence |
| **Cardiovascular** | Cardiovascular | Cardiovascular symptoms; palpitations; erratic heart beats; cardiopulmonary; tachycardia |
| **Mortality** | Mortality | Death; early death; mortality |
| **Ocular** | Ocular | Blurred vision; eye irritation/burning; eye irritation, increased tearing |
| **Renal** | Renal | Acute renal failure; bladder infections; recurrent urinary tract infections; renal disease; oliguric |
| **Miscellaneous** | Miscellaneous | Fever; febrile; methaemoglobinaemia |

*Categorization of angina was based on the respiratory context in which the symptom was reported within the included article.
