# Supplementary material for: A systematic review of human studies assessing the health effects of unburned kerosene-based jet fuels and products across diverse populations and settings
Source: Environ Health. 2026 Mar 16;25:34. doi: 10.1186/s12940-026-01287-7 (PMC13085620; doi:10.1186/s12940-026-01287-7)
Supplement: Supplementary file 9 — Additional File 9. [file 12940_2026_1287_MOESM9_ESM.docx]

| **Author & Year** | **Q1** | **Q2** | **Q3** | **Q4** | **Q5** | **Q6** | **Q7** | **Q8** | **Q9** | **Q10** | **Q11** | **Q12** | **Q13** | **Q14** | **Q15** | **Q16** | **Q17** | **Q18** | **Q19** | **Score Absolute** | **Score (%)** | **Score Quality Category** |  |
| --- | --- | --- | --- | --- | --- | --- | --- | --- | --- | --- | --- | --- | --- | --- | --- | --- | --- | --- | --- | --- | --- | --- | --- |
| Areprekumor et al., 2024 | 1 | 1 | 1 | 0 | 1 | 1 | 0 | 0 | 0 | 1 | 1 | 1 | 1 | 0 | 0 | 0 | 0 | 0 | 0 | 9 | 43 | Fair |  |
| Dayasiri et al., 2017 | 1 | 1 | 1 | 0 | 1 | 0 | 0 | 0 | 0 | 1 | 1 | 1 | 1 | 0 | 0 | 0 | 1 | 1 | 0 | 10 | 48 | Fair |  |
| Dreisbach et al., 2022 | 1 | 1 | 1 | 0 | 0 | 1 | 1 | 0 | 0 | 1 | 1 | 1 | 0 | 0 | 0 | 0 | 1 | 2 | 0 | 11 | 52 | Fair |  |
| Fuente et al., 2019 | 1 | 1 | 1 | 1 | 0 | 1 | 1 | 0 | 0 | 1 | 1 | 1 | 1 | 0 | 1 | 1 | 1 | 1 | 1 | 15 | 71 | Good |  |
| Miko et al., 2023 | 1 | 1 | 1 | 0 | 1 | 0 | 0 | 0 | 0 | 1 | 1 | 1 | 1 | 1 | 0 | 0 | 1 | 0 | 0 | 10 | 48 | Fair |  |
| Oba-Daini et al., 2020 | 1 | 1 | 1 | 0 | 1 | 0 | 0 | 0 | 0 | 1 | 1 | 1 | 1 | 0 | 0 | 0 | 0 | 0 | 0 | 8 | 38 | Poor |  |
| Parekh & Gupta, 2017 | 1 | 1 | 1 | 0 | 1 | 0 | 0 | 1 | 1 | 0 | 1 | 1 | 1 | 0 | 0 | 0 | 1 | 0 | 0 | 10 | 48 | Fair |  |
| Radhakrishnan et al., 2017 | 1 | 1 | 1 | 0 | 1 | 0 | 1 | 0 | 0 | 1 | 1 | 1 | 1 | 0 | 0 | 0 | 1 | 0 | 0 | 10 | 48 | Fair |  |

**Additional file 9. Quality Assessment of included Cross-Sectional Studies.** Scores of each quality assessment item for cross-sectional studies.

The full appraisal tool, including items assessed and corresponding scoring criteria, is provided in Additional file 5.
