# Supplementary material for: A systematic review of human studies assessing the health effects of unburned kerosene-based jet fuels and products across diverse populations and settings
Source: Environ Health. 2026 Mar 16;25:34. doi: 10.1186/s12940-026-01287-7 (PMC13085620; doi:10.1186/s12940-026-01287-7)
Supplement: Supplementary file 10 — Additional File 10. [file 12940_2026_1287_MOESM10_ESM.docx]

**Additional file 10. Quality Assessment of included Case Reports & Case Series.** Scores of each quality assessment item for case reports and case series.

| **Author & Year** | **Q1** | **Q2** | **Q3** | **Q4** | **Q5** | **Q6** | **Q7** | **Score Absolute** | **Score (%)** | **Score Quality Category** |  |
| --- | --- | --- | --- | --- | --- | --- | --- | --- | --- | --- | --- |
| Benítez Riesco et al., 2024 | 1 | 2 | 1 | 1 | 1 | 0 | 1 | 7 | 70 | Good |  |
| Chattopadhyay et al., 2021 | 1 | 2 | 1 | 1 | 1 | 0 | 1 | 7 | 70 | Good |  |
| Contestable, 2017 | 1 | 2 | 1 | 1 | 1 | 1 | 1 | 8 | 80 | Good |  |
| Esashi et al., 2021 | 1 | 2 | 1 | 2 | 1 | 0 | 1 | 8 | 80 | Good |  |
| Fife et al., 2018 | 1 | 2 | 1 | 0 | 0 | 0 | 1 | 5 | 50 | Fair |  |
| Goenka et al., 2022 | 1 | 2 | 1 | 1 | 1 | 1 | 1 | 8 | 80 | Good |  |
| Gupta et al., 2017 | 1 | 2 | 1 | 1 | 1 | 0 | 1 | 7 | 70 | Good |  |
| Guss et al., 2020 | 1 | 2 | 1 | 1 | 1 | 0 | 1 | 7 | 70 | Good |  |
| Hara et al., 2018 | 1 | 2 | 1 | 1 | 1 | 1 | 1 | 8 | 80 | Good |  |
| Kim et al., 2018 | 2 | 2 | 1 | 2 | 1 | 0 | 1 | 9 | 90 | Excellent |  |
| Long & Charles, 2018 | 1 | 2 | 1 | 2 | 1 | 1 | 1 | 9 | 90 | Excellent |  |
| Oreh et al., 2023 | 1 | 2 | 1 | 2 | 1 | 0 | 1 | 8 | 80 | Good |  |
| Poon et al., 2019 | 1 | 2 | 1 | 2 | 1 | 0 | 1 | 8 | 80 | Good |  |
| Ravikanth et al., 2018 | 1 | 2 | 1 | 1 | 1 | 0 | 1 | 7 | 70 | Good |  |
| Salam et al., 2020 | 1 | 2 | 1 | 2 | 1 | 0 | 1 | 8 | 80 | Good |  |
| Sanju et al., 2020 | 2 | 2 | 1 | 2 | 1 | 0 | 1 | 9 | 90 | Excellent |  |

The full appraisal tool, including items assessed and corresponding scoring criteria, is provided in Additional file 6.
