# Supplementary material for: A systematic review of human studies assessing the health effects of unburned kerosene-based jet fuels and products across diverse populations and settings
Source: Environ Health. 2026 Mar 16;25:34. doi: 10.1186/s12940-026-01287-7 (PMC13085620; doi:10.1186/s12940-026-01287-7)
Supplement: Supplementary file 11 — Additional File 11. [file 12940_2026_1287_MOESM11_ESM.docx]

| **Bias Domain** | **Study-Specific Questions** | Heaton et al., 2017 | Dreisbach et al., 2022 | Fuente et al., 2019 |
| --- | --- | --- | --- | --- |
| Selection | Did selection of study participants result in the appropriate comparison groups? | **+** | **-** | **+** |
| Confounding | Did study design or analysis account for important confounding and modifying variables? | **-** | **-** | **--** |
| Attrition/Exclusion | Were outcome data complete without attrition or exclusion from analysis? | **+** | **++** | **++** |
| Detection Bias | Can we be confident in the exposure characterization? | **++** | **-** | **-** |
|  | Can we be confident in the outcome assessment (including blinding of outcome assessors)? | **+** | **+** | **+** |
| Selective Reporting | Were all measured outcomes reported? | **++** | **++** | **++** |
| Other Sources | Were there no other potential threats to internal validity? | **-** | **-** | **+** |

**Additional file 11. Determinations of OHAT Risk of Bias Evaluation for Analytical Studies.** This table outlines risk of bias appraisal questions and results for included analytical studies.

Risk of bias determinations: definitely low risk of bias (++), probably low risk of bias (+), probably high risk of bias (-), or definitely high risk of bias (--).
